# A turn-on fluorescent probe containing a β-ketoester moiety for the selective detection of intracellular hydrazine†

**DOI:** 10.1039/d4ra06525e

**Published:** 2025-01-03

**Authors:** Akira Takagi, Ippei Takashima, Kensuke Okuda

**Affiliations:** a Laboratory of Bioorganic & Natural Products Chemistry, Kobe Pharmaceutical University 4-19-1, Motoyamakita, Higashinada Kobe Hyogo 658-8558 Japan okuda@kobepharma-u.ac.jp; b Institute of Multidisciplinary Research for Advanced Materials, Tohoku University 2-1-1 Katahira, Aoba Sendai Miyagi 980-8577 Japan

## Abstract

Fluorogenic probes containing the β-ketoester structure were developed for selective hydrazine detection. The probe equipped with a cyclopropane moiety, having reduced steric hindrance, showed a higher reaction rate than its dimethyl counterpart. In live cell imaging, the probe detects intracellular hydrazine with minimal cytotoxicity. This study introduces a promising tool for hydrazine detection that may assist in reducing the risks associated with hydrazine exposure in various applications.

## Abstract 2

A selective and quantitative OFF–ON fluorescent hydrazine probe was developed based on a coumarin platform. Elaboration of the β-keto moiety improved the responsiveness of the probe for sensing hydrazine in living cells.

## Graphical abstract

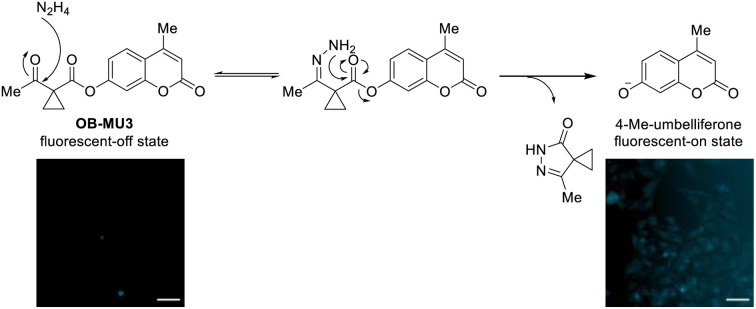

## 1 Introduction

Hydrazine (N_2_H_4_) has long been widely used in many fields as a useful diamine with strong basic, reducing, and nucleophilic properties. Hydrazine can be used as a reducing agent,^1,2^ high-energy rocket propellant,^3^ precursor of pharmaceuticals,^4^ insecticide,^5^ and a raw material for industrial products such as polymers and carbon dioxide sorbents.^6,7^ However, despite its usefulness, hydrazine is highly toxic and is known to be hepatotoxic, neurotoxic, and mutagenic.^8–10^ Its use poses a risk of exposure to the environment during various stages of the manufacturing process. In humans, endogenous aminoacylase may also induce hydrolysis of hydrazine-containing drugs such as isoniazid, releasing hydrazine and/or acetylated hydrazine as a toxic metabolite.^11^ Given the hazardous nature of hydrazine, the sensitive and selective detection of hydrazine in environmental and biological samples is an important issue.

A chemiluminescent probe,^12^ fluorescent probes,^13–15^ and other analytical methods^16–19^ have been developed to detect hydrazine selectively. Among them, fluorescent probes with sufficient solubility, suitable lipophilicity, and negligible toxicity are convenient for assessing hydrazine exposure in living cells, but faster probes with better sensitivity and selectivity are necessary for real applications. Therefore, there has been ongoing development of fluorescent probes that operate on novel detection mechanisms.

**Scheme 1:**
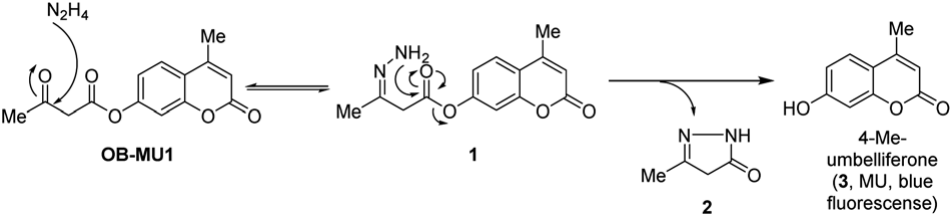
Strategy for selective detection of hydrazine with β-ketoester type fluorescent probe.

We have designed a novel β-ketoester-type fluorescent probe platform (OB-MU1) for detecting hydrazine. The putative mechanism for hydrazine detection is shown in Scheme 1.

First, the ketone moiety of OB-MU1 reacts with hydrazine to form a hydrazone (1). Subsequently, the amine moiety of the hydrazone reacts with the ester in an intramolecular nucleophilic attack to the ester, releasing 5-methyl-2,4-dihydro-3*H*-pyrazol-3-one (2), whereas the fluorophore 4-methylumbelliferone (3) is released and exhibits a fluorescent response. We expected that the combination of the strong nucleophilicity^20^ and the adjacent positioning of the two primary amine moieties within the same molecule could be used to distinguish hydrazine from other bisnucleophiles, including ethylenediamine and hydroxylamine. In addition, strong endogenous mononucleophiles such as ammonia and hydrogen sulfide do not undergo such ring closure reactions due to the unfavorable four-membered ring formation. Levulinic acid ester-type probes^21–25^ have intrinsically fluorogenic reactivity to mononucleophiles by the formation of a five-membered ring as well as to hydrazine by the formation of a six-membered ring. The detection mechanism of OB-MU1 is similar to that of levulinic acid ester-type probes, but our strategy is characterized by five-membered ring formation upon hydrazine detection, giving our probes an advantage.^26^ Another potential research gap with levulinic acid ester-type probes is that it also reacted with sulfite (SO_3_^2−^) to form 2-methyl-5-oxotetrahydrofuran-2-sulfonate by the formation of a five-membered ring and the fluorescent product as seen in resorufin levulinate,^27^ which could complicate hydrazine detection. On the contrary, such a sulfite reaction is unlikely in the case of β-ketoester-type fluorescent probes because unfavorable four-membered ring formation is required for the transformation.

## 2 Results and discussion

**Scheme 2:**
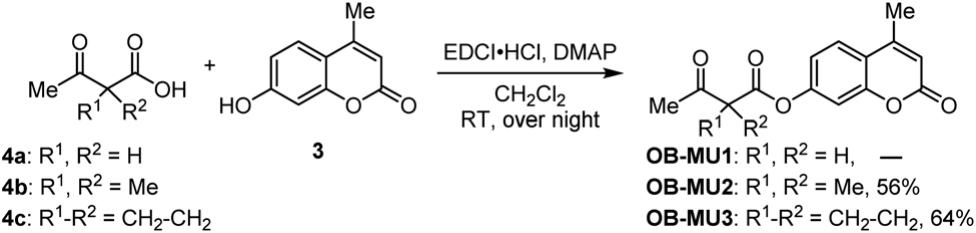
Syntheses of OB-MUs.

First, we attempted to synthesize OB-MU1 by the condensation reaction of 4-methylumbelliferone (3) and 3-oxobutanoic acid (4a), but the desired ester OB-MU1 was not obtained. We attribute this unsuccessful reaction to the reactivity of the active methylene, and condensation with 2,2-disubstituted 3-oxobutanoic acids 4b and 4c gave the desired β-ketoesters OB-MU2 and OB-MU3 (Scheme 2).

**Fig. 1:**
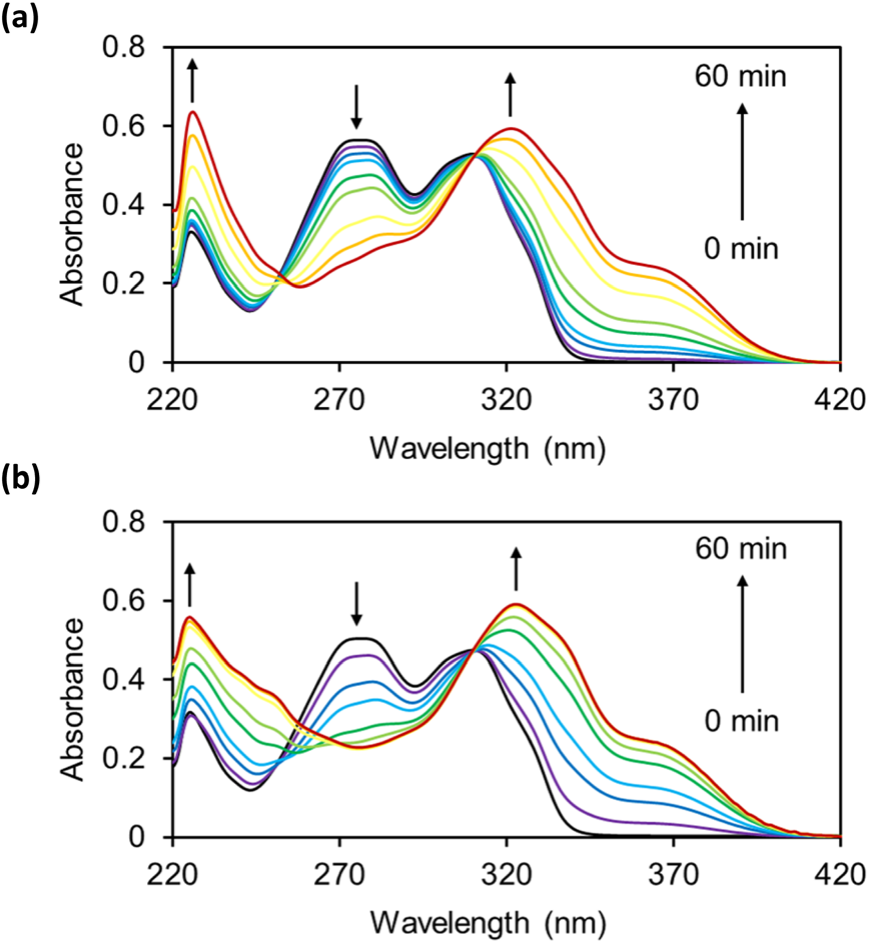
UV-vis spectra of (a) OB-MU2 (50 μM) and (b) OB-MU3 (50 μM) in the presence of hydrazine (1 mM) in 50 mM HEPES buffer (1% acetonitrile, pH 7.4) at 25 °C for 0, 1, 3, 5, 10, 15, 30, 45, and 60 min.

The reactions of OB-MU2 and OB-MU3 (50 μM) with hydrazine (1 mM, 20 eq.) were monitored by UV-vis spectroscopy (Fig. 1) in 50 mM HEPES buffer (pH 7.4). Upon addition of hydrazine, the absorption of the OB-MUs around 270 nm, which is considered to be the maximum absorption of β-ketoesters, decreased in a time-dependent manner. Concomitantly, the absorption peak at around 310 nm, which is considered to be the maximum absorption of esterified 3, shifted to 320 nm, the maximum absorption wavelength of free 3.

**Fig. 2:**
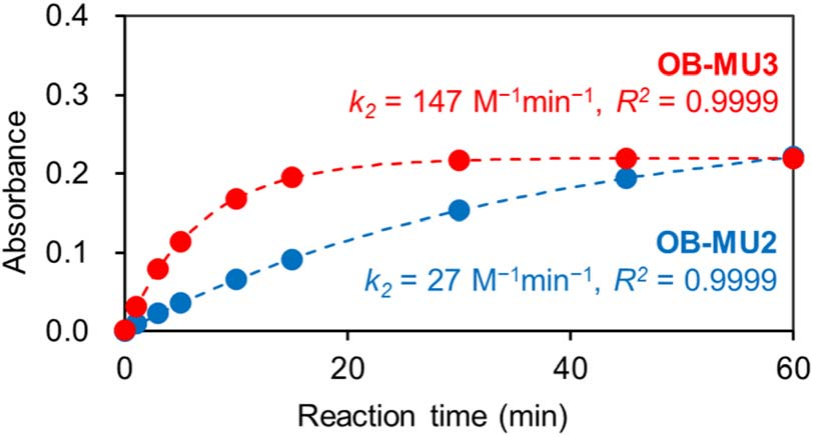
Absorbance plots of OB-MU2 (50 μM, blue circle) and OB-MU3 (50 μM, red circle) in the presence of hydrazine (1 mM) in 50 mM HEPES buffer (1% acetonitrile, pH 7.4) at 25 °C. Absorbance: average of the absorbance at 365–375 nm. Dotted line is calculated absorbance at around 370 nm.

OB-MU2 (apparent *k*_2_ = 27 M^−1^ min^−1^) reacted with hydrazine more slowly than OB-MU3 (apparent *k*_2_ = 147 M^−1^ min^−1^) (Fig. 2).^28,29^ The reaction of OB-MU2 was not complete even after 1 hour, whereas OB-MU3 reacted faster and nearly reached completion by 30 min. Considering that the cyclopropyl moiety is less bulky than the dimethyl moiety, these differences in reaction rates are inconsistent with the Thorpe–Ingold effect for the cyclization reaction; therefore, hydrazone formation with the OB-MUs and hydrazine is likely the rate-determining step.

**Fig. 3:**
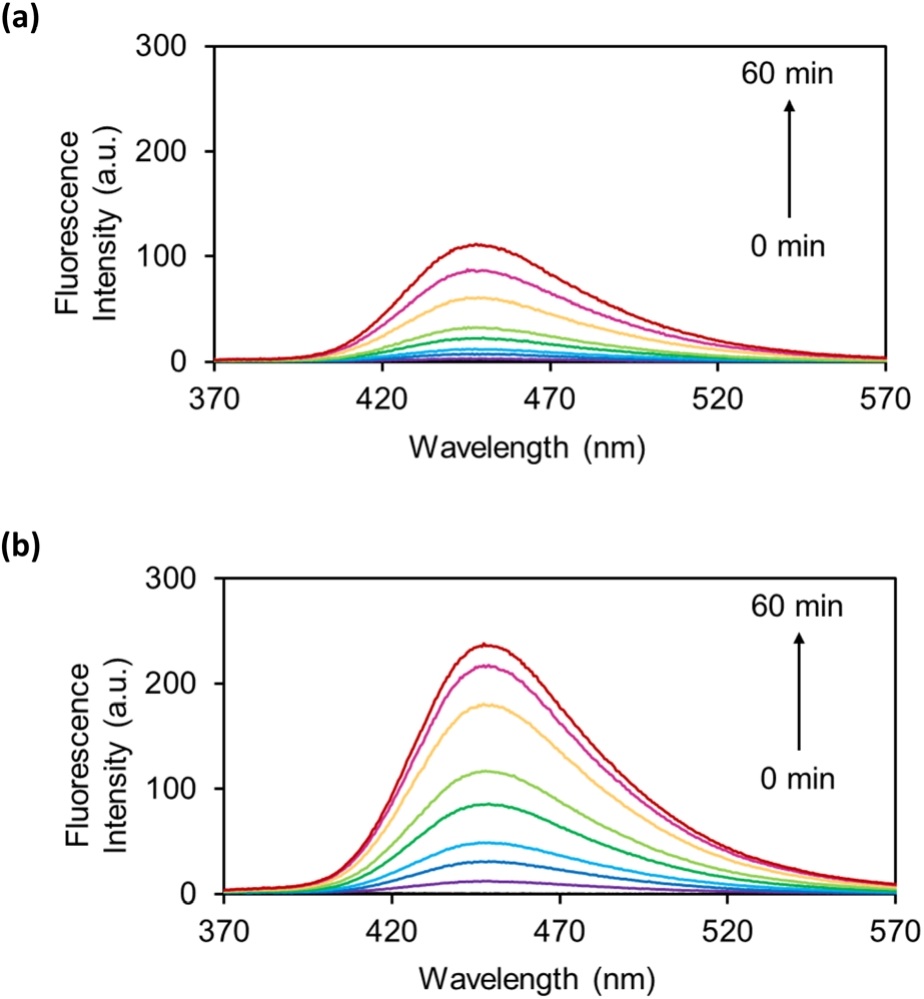
Fluorescence spectra of (a) OB-MU2 (10 μM) and (b) OB-MU3 (10 μM) in the presence of hydrazine (200 μM) in 50 mM HEPES buffer (1% acetonitrile, pH 7.4) at 25 °C for 0, 1, 3, 5, 10, 15, 30, 45, and 60 min (*λ*_ex_: 323 nm, *λ*_em_: 370–570 nm).

The OB-MUs were subsequently evaluated by fluorescence spectroscopy: the reaction of 10 μM OB-MU derivative with 200 μM (20 eq.) hydrazine was observed by measuring the fluorescence spectrum of 3 (Fig. 3).

Fluorescence spectroscopy also revealed a slower reaction of OB-MU2 than OB-MU3 with hydrazine, similar to the UV-vis results. These results indicate that OB-MU3 is a better fluorogenic probe for detecting hydrazine in aqueous environments.

**Fig. 4:**
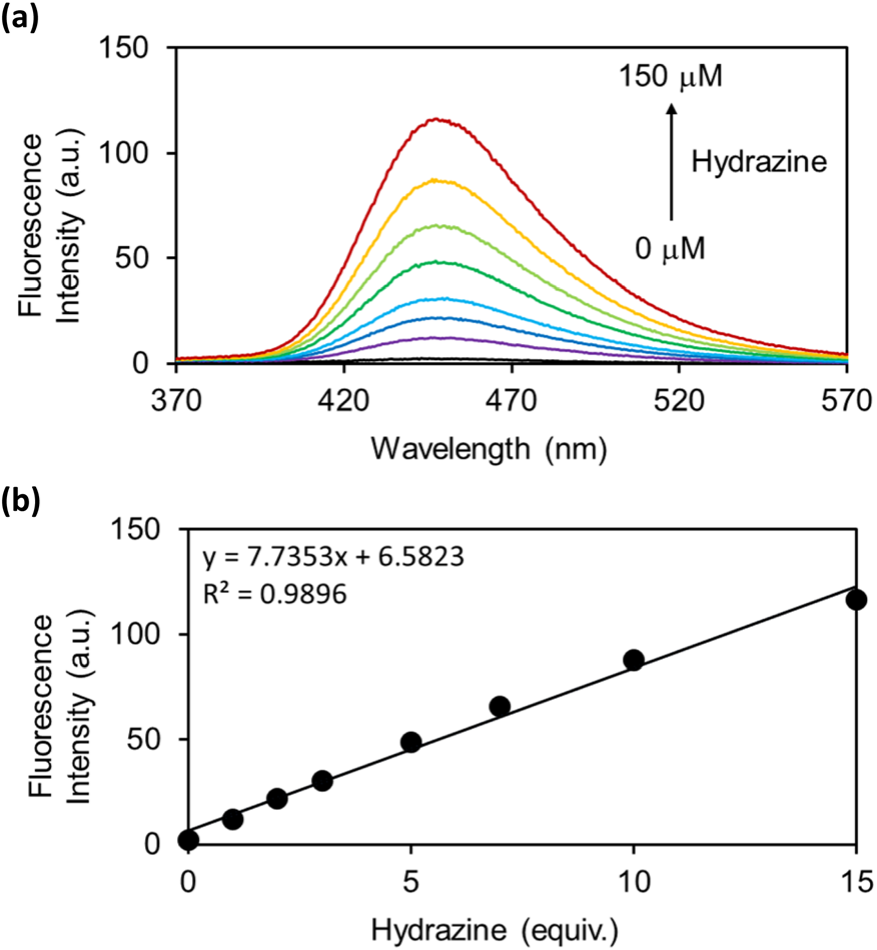
(a) Fluorescence spectra and (b) Plot of fluorescence intensity at 447 nm of OB-MU3 (10 μM) with different equivalents of hydrazine (0, 1, 2, 3, 5, 7, 10, and 15 eq.) in 50 mM HEPES buffer (1% acetonitrile, pH 7.4) at 25 °C, 30 min after addition of hydrazine (*λ*_ex_: 323 nm).

**Fig. 5:**
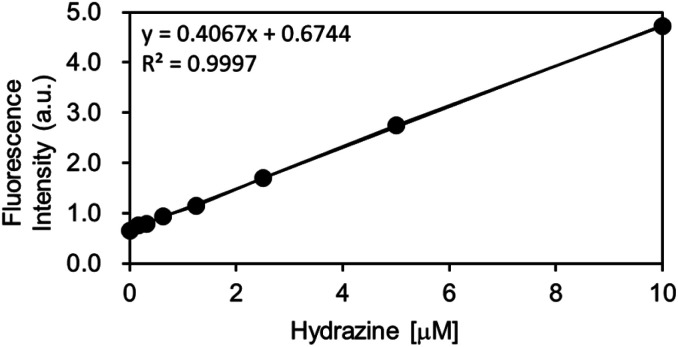
Calibration curve of hydrazine (serial dilution from 10 μM) for OB-MU3 (10 μM) in 50 mM HEPES buffer (pH 7.4, 1% acetonitrile) (*λ*_ex_: 323 nm, *λ*_em_: 447 nm, 25 °C).

As the OB-MU3 probe reacted successfully with hydrazine, further studies were conducted to evaluate its selectivity and other properties. The fluorescence intensity was proportional to the concentration of hydrazine (Fig. 4a and b), indicating that OB-MU3 is a quantitative probe under these conditions. The detection limit of OB-MU3 was found to be 95.3 nM (Fig. 5).

**Fig. 6:**
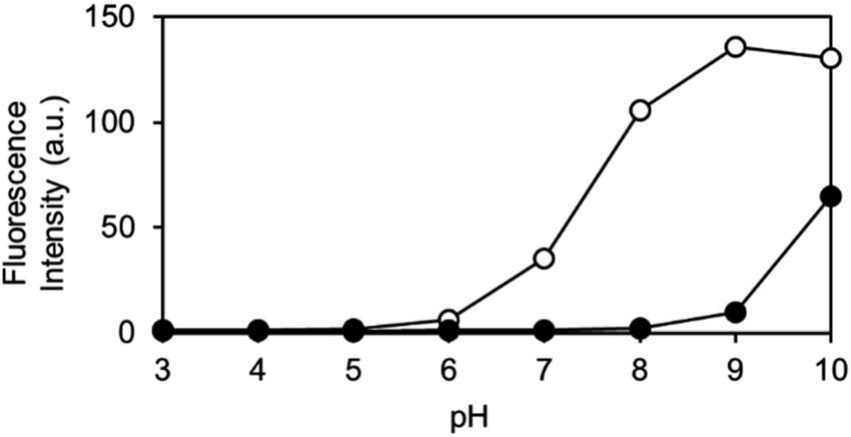
Fluorescent intensity of OB-MU3 (10 μM) under a variety of pH conditions (1% acetonitrile) after 30 min incubation with (200 μM, open circles) or without (0 μM, closed circles) hydrazine (*λ*_ex_: 323 nm, *λ*_em_: 447 nm).

The pH-dependence of the reaction of OB-MU3 with hydrazine was also examined from pH 3 to 10. We observed that under biological conditions (pH 6–8), an increase in pH led to a faster reaction and higher signal in the presence of hydrazine (open circles), whereas nonspecific reactions (closed circles) are still negligible (Fig. 6). These data imply that the pH dependence can be simply derived from the p*K*_a_ of the 3-phenol domain (p*K*_a_ ∼ 7.8), whereas hydrazine and hydrazone intermediates both have sufficient nucleophilicity when the pH is above 5, according to the p*K*_a_s of their conjugated acids. Above pH 8, the fluorogenic reaction by non-specific alkaline hydrolysis competes with the intended reaction with hydrazine.

**Fig. 7:**
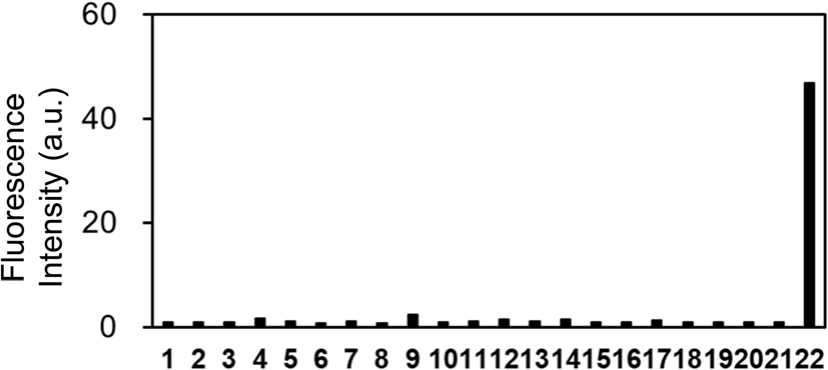
Fluorescence response of OB-MU3 (10 μM) towards different analytes (200 μM) at 25 °C, 30 min after addition of analytes in 50 mM HEPES buffer (1% acetonitrile, pH 7.4) (*λ*_ex_: 323 nm, *λ*_em_: 447 nm). Analyte 1: blank, 2: ammonia, 3: hydroxylamine, 4: ethylenediamine, 5: S^2−^, 6: aniline, 7: methylamine, 8: piperidine, 9: *p*-tolylhydrazine, 10: lysine, 11: glycine, 12: SO_3_^2−^, 13: S_2_O_3_^2−^, 14: Cu^+^, 15: Cu^2+^, 16: Zn^2+^, 17: Fe^2+^, 18: Fe^3+^, 19: Mn^2+^, 20: Ni^2+^, 21: Co^2+^, 22: hydrazine.

Further examination of the selectivity of OB-MU3 toward different test substances showed that the fluorescence response to various amines, amino acids, reducing substances, and metal ions was weak (Fig. 7 and S1†). As anticipated, the better selectivity of OB-MU3 for hydrazine compared to other nucleophiles including sulfite (#12) is thought to be due to the formation of a five-membered ring with two adjacent nucleophilic amines.

**Scheme 3:**
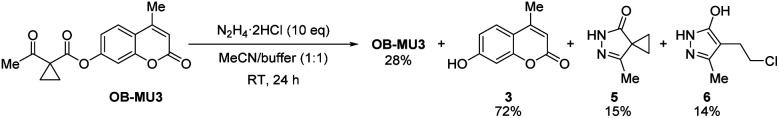
Analysis of the reaction of OB-MU3 with hydrazine.

**Scheme 4:**
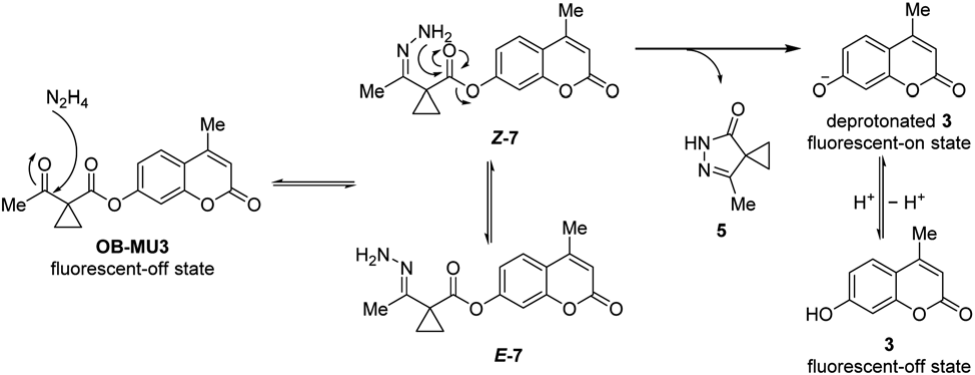
Plausible fluorogenic reaction mechanism of OB-MU3 with hydrazine.

To confirm the fluorogenic reaction mechanism of OB-MU3 with hydrazine, we evaluated the product formed from the reaction when hydrazine was added to OB-MU3 on a 1 mmol scale. The addition of 10 equivalents of hydrazine dihydrochloride to OB-MU3 in an aqueous acetonitrile solution yielded 3 in quantities corresponding to OB-MU3 consumption, as well as formation of what is presumably the cyclized product 5 which likely reacts with HCl to form 6 (Scheme 3). These results suggest that the reaction of OB-MU3 with hydrazine proceeded as we expected in Scheme 1. The plausible reaction mechanism of fluorogenic OB-MU3 with hydrazine is shown in Scheme 4. First, the ketone moiety of OB-MU3 reacts with hydrazine to form a hydrazone (7). 7 exists as either the *E*-7 or the *Z*-7 isomer. Subsequently, the amine moiety of *Z*-7 reacts with the ester in an intramolecular nucleophilic attack to the ester, releasing 5, while the fluorophore 3 is released as the phenolate and exhibits a fluorescent response. We also state here that 6 with reactive alkyl halide may appear toxic similar to the HaloTag due to its possible modification of intracellular nucleophiles such as glutathione. However, generation of potentially toxic 6 under physiological conditions is unlikely because the chloride ion concentration is low (5 to 60 mM)^30^ compared to the concentrations found in our experiment (2.0 M) which comes from the use of the stable dihydrochloride form of hydrazine.

**Fig. 8:**
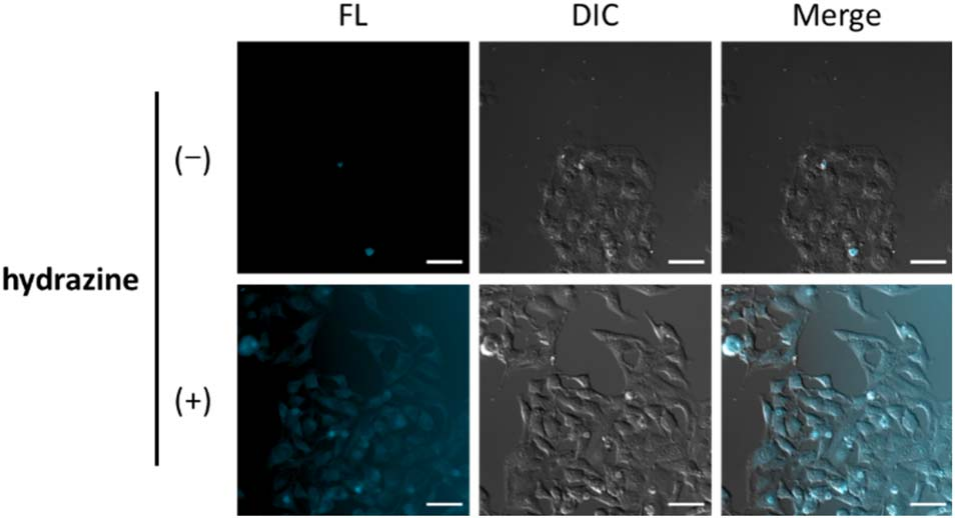
Fluorescence images of HeLa cells in the UV channel (excitation 370–400 nm, EM filter 410–440 nm, FL) and the DIC images. The cells were incubated with 20 μM OB-MU3 for 30 min followed by 0 or 600 μM hydrazine for 30 min; scale bar: 50 μm.

**Fig. 9:**
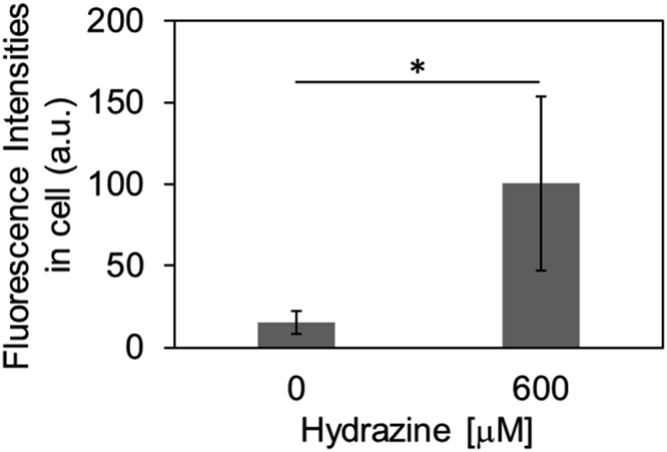
The fluorescence intensities in intracellular region after the treatment of OB-MU3. The error bar is the standard deviation (*n* = 5). The asterisk presents significant difference in the statistical assessment using Student's *t*-test (*p* < 0.05).

**Fig. 10:**
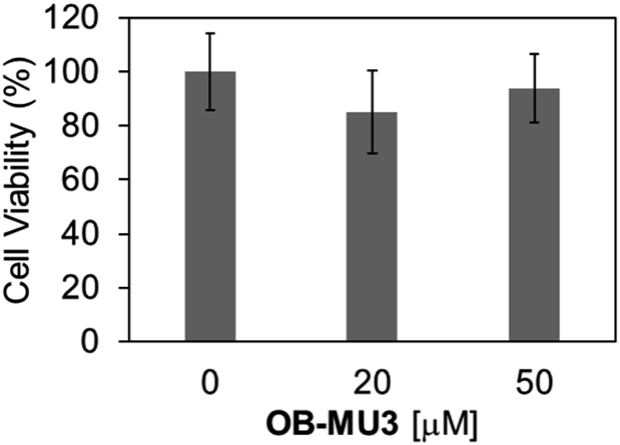
Cell viabilities of HeLa cells after treatment of 0–50 μM OB-MU3 for 1 hour in humidified incubator at 37 °C with 5% CO_2_. The absorbance at 450 nm and 650 nm was obtained by the treatment of WST-8 assay reagent for 4 hours. The obtained value was normalized with that in non-treated cells ([OB-MU3] = 0 μM) to calculate the percentage of cell viability. The error bar is the standard deviation (*n* = 4).

Finally, we evaluated the ability of OB-MU3 to detect hydrazine in live-cell imaging using fluorescence microscopy. After HeLa cells were treated with 20 μM OB-MU3 and washed with Hanks' balanced salt solution (HBSS)(+), they were exposed to HBSS(+) containing 600 μM hydrazine (Fig. 8). Fluorescence imaging results showed that after treatment with hydrazine, an OB-MU3-derived signal was observed throughout the cell with a fluorescence increase of *ca.* 7-fold (Fig. 9). Therefore, OB-MU3 is a hydrazine probe based on a novel cyclization reaction that can visualize exogenous hydrazine in live cells. Finally, we confirmed that up to 50 μM of OB-MU3 exhibited minimal acute cell toxicity (Fig. 10) and it is thought that OB-MU3 exerts a negligible influence on cells at low concentrations (20 μM). We plan to exchange 3 with a long-wavelength fluorophore like TokyoGreen for further biological applications and/or imaging within specific organelles using the same synthesis scheme as that used for OB-MU3.

## 3 Conclusions

In summary, we have developed novel fluorescent probes bearing the β-ketoester structure, OB-MU2 and OB-MU3, for the detection of hydrazine. Based on UV-vis and fluorescence spectroscopic measurements, the cyclopropyl moiety of OB-MU3 accelerates the response to hydrazine compared to the dimethyl structure of OB-MU2. OB-MU3 also exhibited a fluorogenic response under aqueous conditions containing 1% organic solvent (acetonitrile) at physiological pH, with up to a 54-fold increase in fluorescence. In addition, OB-MU3 demonstrated a very strong hydrazine-selective response, showing little reaction to many nucleophiles and reducing agents, with hydroxylamine and hydrogen sulfide as notable examples. Moreover, OB-MU3 can visualize intracellular hydrazine. Coumarin-based OB-MU3, which has short excitation and emission wavelengths, has drawbacks for bioimaging applications such as limited imaging depth and interference from the autofluorescence from biological substances.^31^ The moderate signal-to-noise ratio of OB-MU3 (Fig. 8 and 9) was caused by low intracellular retention of released 3. Therefore, the signal-to-noise ratio can be improved by replacing 3 with a hydrophilic fluorophore with better intracellular retention. In the future, β-ketoester structures will be combined with long-wavelength fluorescent dyes that are more suited than 7-hydroxycoumarin for bioimaging applications to develop probes that can similarly detect intracellular hydrazine with high sensitivity.

## 4 Experimental

### 4.1. General information for synthetic procedures

Reagents: all reactions were carried out under an inert atmosphere in a round bottom flask containing a stir-bar with a rubber septum except as noted otherwise. Anhydrous dichloromethane (CH_2_Cl_2_) was purchased from FUJIFILM Wako Pure Chemical Co. and used without further purification. All other reagents were purchased from Tokyo Chemical Industry Co., Nacalai Tesque Inc., or FUJIFILM Wako Pure Chemical Co. and used without further purification. SiliaFlash^®^ F60, 40–63 μm, #R10030B (Silicycle Inc., Quebec, Canada) or Chromatorex PSQ60B (Fuji Silysia Chemical Ltd., Kasugai, Japan) was used for silica gel flash chromatography.

### 4.2. Analytical methods

All reactions were monitored by thin-layer chromatography with E. Merck silica gel 60 F_254_ pre-coated plates (0.25 mm) and were visualized by UV (254 nm). IR spectra were obtained on a PerkinElmer Spectrum One. ^1^H NMR and ^13^C NMR spectra were recorded on a JEOL ECZ400S spectrometer (^1^H: 400 MHz, ^13^C: 100 MHz) instrument. Chemical shifts are reported in ppm relative to the carbons of deuterated solvents (CDCl_3_: 77.0 ppm, DMSO-*d*_6_: 39.5 for ^13^C) or the internal standard tetramethylsilane (CDCl_3_ and DMSO-*d*_6_: 0.00 ppm for ^1^H). The mass spectra were measured on a Thermo Fisher Scientific LTQ Orbitrap Discovery. Melting points were determined with a Yanaco micro melting point apparatus MP-J3. Yields refer to isolated yields of compounds greater than 95% purity as determined by ^1^H NMR analysis. All new products were characterized by ^1^H NMR, ^13^C NMR, IR, and HRMS. UV-vis spectroscopy was recorded by Cary 8454 (Agilent). Fluorescence spectroscopy was recorded by Duetta (HORIBA) and SpectraMax iD5 multiplate reader (Molecular Devices).

### 4.3. Procedure for synthesis of probes

#### 4.3.1 Synthesis of OB-MU2

To a solution of 2,2-dimethyl-3-oxobutanoic acid^32^ (4b, 260 mg, 2.00 mmol) in CH_2_Cl_2_ (4.0 mL), 4-methylumbelliferone (3, 177 mg, 1.00 mmol), 1-ethyl-3-(3-dimethylaminopropyl)carbodiimide hydrochloride (EDCI·HCl, 560 mg, 2.92 mmol), and 4-dimethylaminopyridine (DMAP, 13.0 mg, 0.106 mmol) were added and stirred for 14 hours at room temperature. Reaction mixture was quenched by adding H_2_O and extracted with CHCl_3_. The aqueous layer was extracted with CHCl_3_ twice. The combined organic layers were dried over anhydrous Na_2_SO_4_ and filtered and solvents were removed under reduced pressure. The residue was purified by column chromatography (SiO_2_, CHCl_3_) to obtain OB-MU2 (162 mg, 56%) as a colorless solid. Rf: (CHCl_3_/MeOH = 30 : 1): 0.63. Mp: 90–91 °C. ^1^H NMR (400 MHz, CDCl_3_) *δ*: 7.62 (d, *J* = 8.0 Hz, 1H), 7.11 (d, *J* = 2.5 Hz, 1H), 7.06 (dd, *J* = 8.0, 2.5 Hz, 1H), 6.29 (d, *J* = 1.5 Hz, 1H), 2.44 (d, *J* = 1.5 Hz, 3H), 2.31 (s, 3H), 1.55 (s, 6H). ^13^C NMR (100 MHz, CDCl_3_) *δ*: 205.1, 171.5, 160.2, 154.0, 152.8, 151.8, 125.5, 117.9, 117.6, 114.5, 110.0, 56.1, 25.5, 21.7, 18.6. IR (KBr) 3073, 2976, 1761, 1726, 1709, 1616 cm^−1^. HRMS (ESI) *m*/*z* calcd for [C_16_H_16_O_5_ + Na^+^] 311.0890, found 311.0884.

#### 4.3.2 Synthesis of OB-MU3

To a solution of 1-acetylcyclopropane-1-carboxylic acid^33^ (4c, 609 mg, 4.75 mmol) in CH_2_Cl_2_ (20 mL), 3 (351 mg, 1.99 mmol), EDCI·HCl (933 mg, 4.87 mmol), and DMAP (16.8 mg, 0.138 mmol) were added and stirred for 19 hours at room temperature. Reaction mixture was quenched by adding H_2_O and extracted with CHCl_3_. The aqueous layer was extracted with CHCl_3_ twice. The combined organic layers were dried over anhydrous Na_2_SO_4_ and filtered and solvents were removed under reduced pressure. The residue was purified by column chromatography (SiO_2_, CHCl_3_) to obtain OB-MU3 (364 mg, 64%) as a colorless solid. Rf: (CHCl_3_/MeOH = 30 : 1): 0.59. Mp: 129–130 °C. ^1^H NMR (400 MHz, CDCl_3_) *δ*: 7.63 (d, *J* = 8.5 Hz, 1H), 7.12 (d, *J* = 2.5 Hz, 1H), 7.07 (dd, *J* = 8.5, 2.5 Hz, 1H), 6.30 (d, *J* = 1.5 Hz, 1H), 2.58 (s, 3H), 2.45 (d, *J* = 1.5 Hz, 3H), 1.78–1.70 (m, 4H). ^13^C NMR (100 MHz, CDCl_3_) *δ*: 201.8, 169.2, 160.3, 154.2, 152.5, 151.8, 125.5, 118.1, 117.8, 114.7, 110.3, 34.9, 29.9, 20.4, 18.7. IR (KBr) 3077, 1752, 1730, 1700, 1612 cm^−1^. HRMS (ESI) *m*/*z* calcd for [C_16_H_14_O_5_ + H^+^] 287.0914, found 287.0911.

### 4.4. Analysis of the reaction of OB-MU3 with hydrazine

To a solution of OB-MU3 (282 mg, 0.985 mmol) in acetonitrile (5 mL) and sodium phosphate buffer (100 mM, pH = 7.5, 5 mL), hydrazine dihydrochloride (1.04 g, 9.91 mmol) was added and stirred for 24 hours at room temperature. Reaction mixture was diluted with EtOAc and washed with aqueous NaHCO_3_. The aqueous layer was extracted with EtOAc twice. The combined organic layers were dried over Na_2_SO_4_ and filtered and solvents were removed under reduced pressure. The residue was purified by column chromatography (SiO_2_, CHCl_3_/MeOH = 10 : 1 to 4 : 1) to give the unreacted OB-MU3 (78.4 mg, 28%), concomitant with fluorescent 3 (125.5 mg, 72%) as a colorless solid, 5 (19.1 mg, 15%) as a colorless solid, and 6 (22.1 mg, 14%) as a colorless solid.

#### 4.4.1 7-Methyl-5,6-diazaspiro[2.4]hept-6-en-4-one (5)^34^

Rf: (CHCl_3_/MeOH = 10 : 1): 0.58. Mp: 136–138 °C (lit. 140–141 °C).^34^^1^H NMR (400 MHz, DMSO-*d*_6_) *δ*: 11.1 (s, 1H), 1.80 (s, 3H), 1.70 (q, *J* = 4.0 Hz, 2H), 1.35 (q, *J* = 4.0 Hz, 2H). ^13^C NMR (100 MHz, DMSO-*d*_6_) *δ*: 176.1, 159.3, 31.4, 17.0 (2C), 12.3. HRMS (ESI) *m*/*z* calcd for [C_6_H_8_ON_2_ + Na^+^] 147.0523, found 147.0523.

#### 4.4.2 4-(2-Chloroethyl)-3-methyl-1*H*-pyrazol-5-ol (6)^34^

Rf: (CHCl_3_/MeOH = 10 : 1): 0.23. Mp: 167–168 °C (lit. 170–171 °C).^34^^1^H NMR (400 MHz, DMSO-*d*_6_) *δ*: 10.44 (br s, 1H), 3.57 (t, *J* = 7.5 Hz, 2H), 2.63 (t, *J* = 7.5 Hz, 2H), 2.06 (s, 3H). ^13^C NMR (100 MHz, DMSO-*d*_6_) *δ*: 159.7, 137.6, 97.2, 44.5, 25.6, 9.9. HRMS (ESI) *m*/*z* calcd for [C_6_H_9_ON_2_^35^Cl + H^+^] 161.0476, found 161.0477, calcd for [C_6_H_9_ON_2_^37^Cl + H^+^] 163.0447, found 163.0447.

### 4.5. Absorption and fluorescence measurement of probes

#### 4.5.1 Time dependent UV-vis spectra of probes treated with hydrazine

To a quartz cuvette, 2.94 mL of 50 mM HEPES buffer (pH 7.4), 30 μL of OB-MU2 or OB-MU3 (5 mM solution in acetonitrile, final conc. 50 μM), and 30 μL hydrazine (100 mM solution in H_2_O, final conc. 1 mM) were added and incubated for each time (0, 1, 3, 5, 10, 15, 30, 45, and 60 min) at 25 °C. After incubation, UV-vis spectra were measured using a Cary 8454 spectrophotometer (*λ*_abs_: 210–1100 nm, 25 °C).

#### 4.5.2 Kinetics analysis

All kinetics analyses were conducted with the following eqn (1) and (2) according to the literatures,^28,29^1
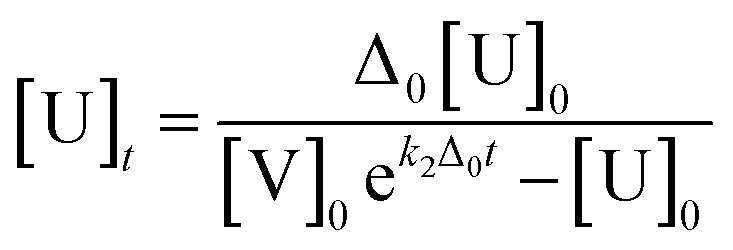

2 [P]_*t*_ = [U]_0_ − [U]_*t*_

* Δ_0_ = [V]_0_ − [U]_0_, [V]_0_ = 20[U]_0_, *k*_2_: second order rate constant [M^−1^ min^−1^], U: hydrazine probe (OB-MU2 or OB-MU3), V: hydrazine, P: product (4-methylumbelliferone)

The estimated absorbance (*A*_365–375_: average of absorbance at 365–375 nm) was calculated with [U]_*t*_ and [P]_*t*_ given by eqn (1) and (2), respectively, and the absorption constants (average of extinction coefficients at 365–375 nm). The apparent *k*_2_ was obtained by least squares curve fitting between the measured and estimated absorbance with scanning of *k*_2_ value in eqn (1).

#### 4.5.3 Time dependent fluorescence spectra of probes treated with hydrazine

To a quartz cuvette, 2.94 mL of 50 mM HEPES buffer (pH 7.4), 30 μL of OB-MU2 or OB-MU 3 (1 mM solution in acetonitrile, final conc. 10 μM), and 30 μL hydrazine (20 mM solution in H_2_O, final conc. 200 μM) were added and incubated for each time (0, 1, 3, 5, 10, 15, 30, 45, and 60 min) at 25 °C. After incubation, fluorescence spectra were measured using a Duetta fluorescence spectrometer (*λ*_ex_: 323 nm, *λ*_em_: 370–570 nm, 25 °C).

#### 4.5.4 Concentration dependent fluorescence spectra of OB-MU3 treated with hydrazine

To a quartz cuvette, 2.94 mL of 50 mM HEPES buffer (pH 7.4), 30 μL of OB-MU3 (1 mM solution in acetonitrile, final conc. 10 μM), and 30 μL hydrazine (0, 1, 2, 3, 5, 7, 10, or 15 mM solution in H_2_O, final conc. 0, 10, 20, 30, 50, 70, 100, 150 μM) were added and incubated for 30 min at 25 °C. After incubation, fluorescence spectra were measured using a Duetta fluorescence spectrometer (*λ*_ex_: 323 nm, *λ*_em_: 370–570 nm, 25 °C).

Additionally, using the following equation DL = *K* × Sb_1_/*S*; the detection limit (DL) of OB-MU3 (10 μM) by fluorescence (*λ*_ex_: 323 nm, *λ*_em_: 447 nm, 25 °C) for hydrazine (serial dilution from 10 μM) in 50 mM HEPES buffer (pH 7.4, 1% acetonitrile) was calculated, where *K* = 3; Sb_1_ is the standard deviation of the blank solution; and *S* is the slope of the calibration curve. Sb_1_ = 0.01292, *S* = 0.4067, ∴DL = 95.3 nM.

#### 4.5.5 pH-Dependent fluorescence intensity of OB-MU3 treated with hydrazine

To each well of a flat bottom black 96-well plate (Evergreen, #290-8195-Z1F), 196 μL of buffer (Mcllvaine buffer for pH 3.0, 4.0, 5.0, 6.0, 7.0, and 8.0; 100 mM sodium borate buffer for pH 9.0 and 10.0), 2 μL of OB-MU3 (1 mM solution in acetonitrile, final conc. 10 μM), and 2 μL of hydrazine (0 or 20 mM solution in H_2_O, final conc. 0 or 200 μM) were added, followed by incubation for 30 min at 25 °C. After incubation, fluorescence intensity was measured by SpectraMax iD5 multiplate reader (*λ*_ex_: 323 nm, *λ*_em_: 447 nm, 25 °C).

#### 4.5.6 Fluorescence selectivity of analytes

To each well of a flat bottom black 96-well plate (Evergreen, #290-8195-Z1F), 196 μL of 50 mM HEPES buffer (pH 7.4), 2 μL of OB-MU3 (1 mM in acetonitrile, final conc. 10 μM), and 2 μL of each analyte (for 1: H_2_O, 2: ammonia, 3: NH_2_OH·HCl, 4: ethylenediamine, 5: Na_2_S, 6: aniline, 7: methylamine, 8: piperidine, 9: *p*-tolylhydrazine, 10: lysine, 11: glycine, 12: Na_2_SO_3_, 13: Na_2_S_2_O_3_, 14: CuBr, 15: CuBr_2_, 16: ZnSO_4_, 17: FeSO_4_, 18: FeCl_3_, 19: MnCl_2_, 20: NiCl_2_, 21: CoCl_2_, 22: N_2_H_4_·2HCl, 20 mM in H_2_O, final conc. 200 μM) were added and incubated for 20 and 30 min at 25 °C. After incubation, fluorescence intensity was measured using a SpectraMax iD5 multiplate reader (*λ*_ex_: 323 nm, *λ*_em_: 447 nm, 25 °C).

### 4.6. Cellular experiments

#### 4.6.1 Cell culture

HeLa cells were cultured in Dulbecco's modified Eagle's medium (DMEM, 4.5 g per L glucose; Nacalai Tesque Inc., #08458-45) supplemented with 5% fetal bovine serum (FBS; Sigma Lot No. S.15N348), 50 μg per mL kanamycin sulfate (Meiji Seika Pharma Co.), 50 U per mL penicillin G potassium (Meiji Seika Pharma Co.), and 50 μg per mL streptomycin sulfate (Meiji Seika Pharma Co.) at 37 °C under a humidified atmosphere of 5% CO_2_ in air. Cell passages from subconfluent cultures were performed once a week using a trypsin–ethylenediaminetetraacetic acid (EDTA) solution (10 times diluted FUJIFILM Wako Pure Chemical Co., #208-17251).

#### 4.6.2 Fluorescence imaging using an microscope AxioObserver 7

For fluorescence bioimaging, cells (5.0 × 10^4^ cells per mL) were cultured in 500 μL DMEM for 24 hours in each compartment with a 35 mm glass-bottomed dish (CellVIEW, 4 compartments, sterile, Greiner bio-one, #627870). After washing twice with 500 μL HBSS(+), the cells were incubated with phenylmethylsulfonyl fluoride (PMSF, 2 mM) in 250 μL HBSS(+) and OB-MU3 (40 μM) in 250 μL HBSS(+) for 30 min. After washing once with 500 μL HBSS(+), the cells were treated with 0 or 600 μM hydrazine dihydrochloride in 500 μL HBSS(+) for 30 min, followed by observation of the cells using an AxioObserver 7 inverted microscope (Carl Zeiss AG) equipped with 20× (N.A. 0.8) objective lens, Colibri7 LED illumination system, and Prime BSI sCMOS camera (Teledyne Photometrics) under differential interreference contrast (DIC) and fluorescent mode (fluorescence channel: *λ*_ex_ = 370–400 nm, *λ*_em_ = 410–440 nm). We also confirmed that no fluorescence was observed without the OB-MU3 probe for this channel with or without hydrazine dihydrochloride (data not shown). All treatments were conducted in CO_2_ incubator (37 °C, 5% CO_2_, humidified atmosphere). PMSF (#160-12183) was purchased from FUJIFILM Wako Pure Chemical Co. HBSS(+) was purchased from FUJIFILM Wako Pure Chemical Co. (#084-08965) or Nacalai Tesque Inc. (#09735-75).

#### 4.6.3 Cell viability check after the incubation with OB-MU3

Cells (1.0 × 10^5^ cells per mL) were cultured in 100 μL DMEM with 5% FBS for 24 hours in each well (TPP, cell culture 96-well plate, flat bottom, clbt, #92696). After removal of the medium *via* aspiration and washing with PBS(−) (100 μL), the cells were incubated in HBSS(+) containing different concentrations [OB-MU3] = 0, 20, and 50 μM for 1 hour. After removal of the solution, the cells were incubated in 100 μL DMEM with 5% FBS containing 10% WST-8 cell counting solution (Kishida Chemical Co. Ltd, #260-96160). After 4 hours of treatment, absorbance was measured at 450 nm (Abs_450_) to quantify the metabolite water-soluble formazan and at 650 nm (Abs_650_) to measure background absorbance using a SpectraMax iD5 multiplate reader. Cell viability was calculated from the mean values of four wells using the following equation:
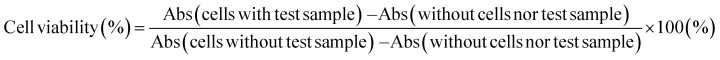
Abs = Abs_450_ − Abs_650_.

## Data availability

The data underlying this study are available in the published article and its ESI.†

## Author contributions

KO conceived the project. All authors designed the experiments. AT and KO performed the experiments. All authors analyzed the data and contributed to manuscript preparation and revision.

## Conflicts of interest

There are no conflicts to declare.

## Supplementary Material

RA-015-D4RA06525E-s001
