# Supplementary material for: A turn-on fluorescent probe containing a β-ketoester moiety for the selective detection of intracellular hydrazine
Source: RSC Adv. 2025 Jan 3;15(1):428–34. doi: 10.1039/d4ra06525e (PMC11697295; doi:10.1039/d4ra06525e)
Supplement: RA-015-D4RA06525E-s001 [file RA-015-D4RA06525E-s001.pdf]

Supplementary Information (SI) for RSC Advances.
This journal is © The Royal Society of Chemistry 2025

Supplementary Information

A turn-on fluorescent probe containing a β-ketoester moiety for the selective detection of intracellular hydrazine

Akira Takagi, Ippei Takashima,[†] and Kensuke Okuda*
Laboratory of Bioorganic & Natural Products Chemistry, Kobe Pharmaceutical University, 4-19-1, Motoyamakita, Higashinada, Kobe, Hyogo 658-8558, Japan
† Present address: Institute of Multidisciplinary Research for Advanced Materials, Tohoku University, 2-1-1 Katahira, Aoba, Sendai, Miyagi 980-8577, Japan.

* Correspondence phone/facsimile: +81-78-441-7545, e-mail: okuda@kobepharma-u.ac.jp (K.O.)

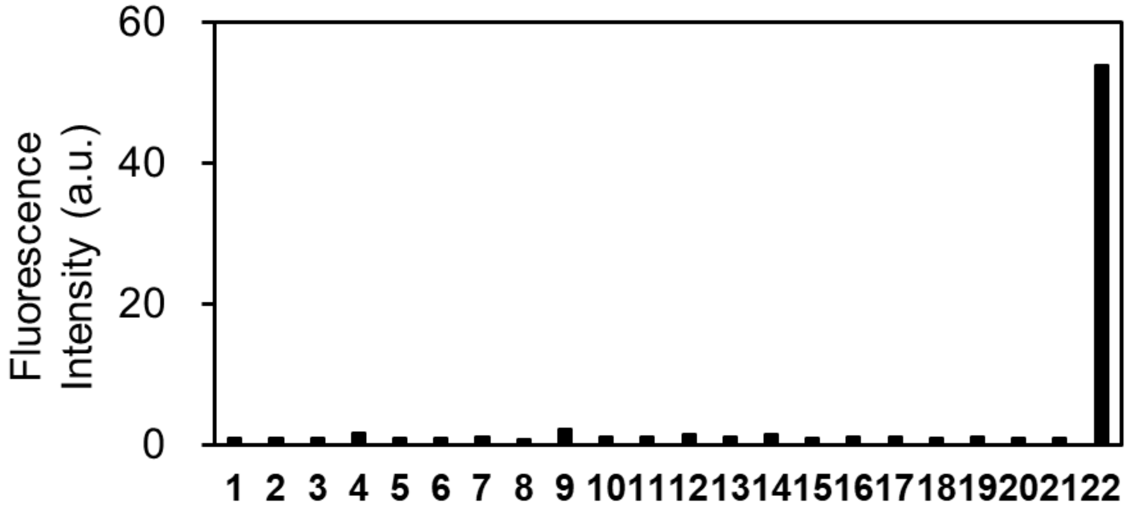

Fig. S1. Fluorescence response of **OB-MU3** (10 μM) towards different analytes (200 μM) at 25 °C, 20 min after addition of analytes in 50 mM HEPES buffer (1% acetonitrile, pH 7.4) (λ_{ex}: 323 nm, λ_{em}: 447 nm). Analyte 1: blank, 2: ammonia, 3: hydroxylamine, 4: ethylenediamine, 5: S^{2-}, 6: aniline, 7: methylamine, 8: piperidine, 9: *p*-tolylhydrazine, 10: lysine, 11: glycine, 12: SO_3^{2-}, 13: $S_2O_3^{2-}$, 14: Cu^{+}, 15: Cu^{2+}, 16: Zn^{2+}, 17: Fe^{2+}, 18: Fe^{3+}, 19: Mn^{2+}, 20: Ni^{2+}, 21: Co^{2+}, 22: hydrazine.

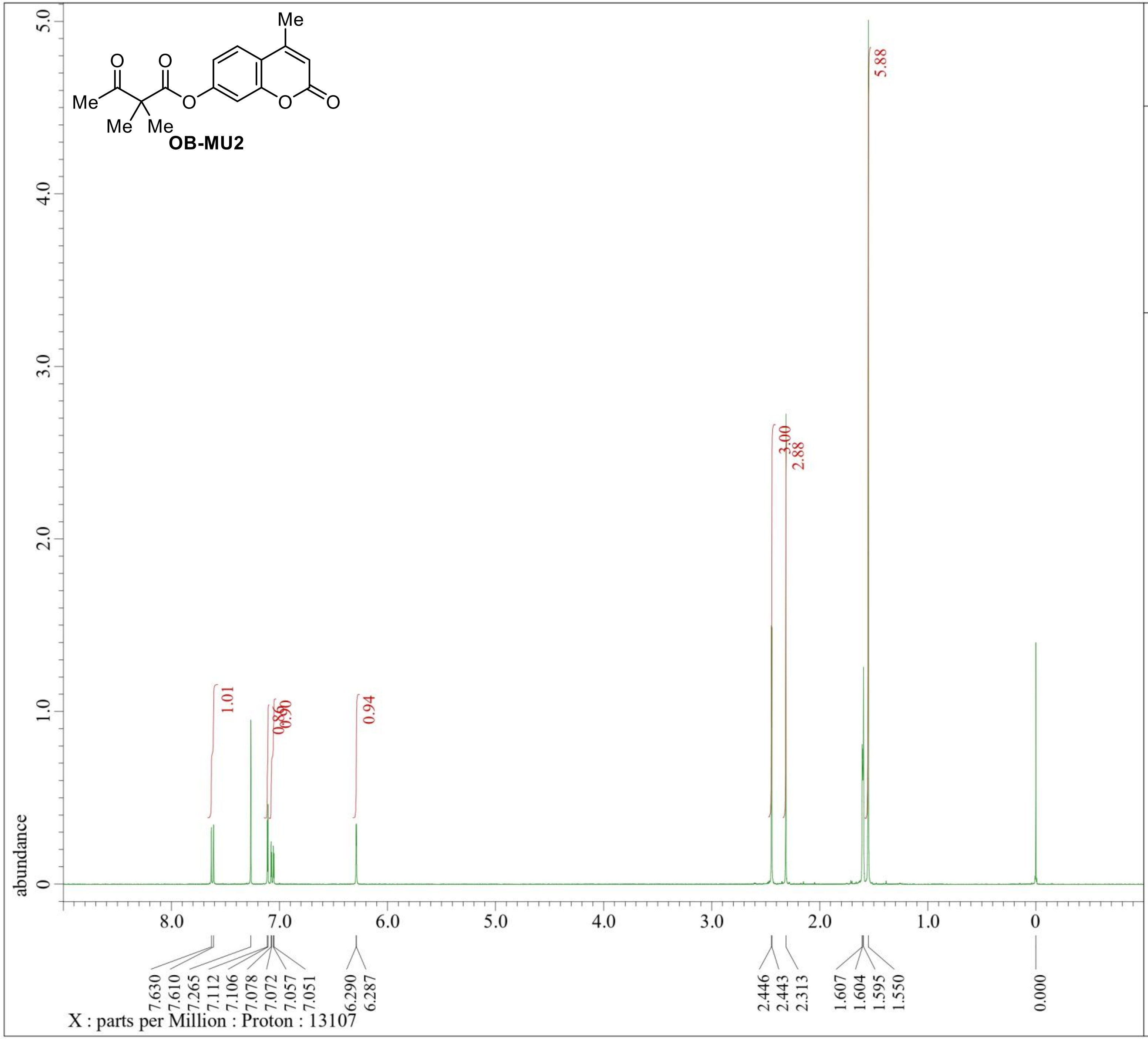

JEOL

```
---- PROCESSING PARAMETERS ----
sexp( 0.2[Hz], 0.0[s] )
trapezoid( 0[%], 0[%], 80[%], 100[%] )
zerofill( 1, TRUE )
fft( 1, TRUE, TRUE )
machinephase
ppm
phase( -0.15555, 1.79469, 72.95895[%] )
```

```
Filename            = 16ATA2-65-2_Proton-1-
Author              = delta
Experiment          = proton_auto.jxp
Sample_Id           = 16ATA2-65-2
Solvent             = CHLOROFORM-D
Actual_Start_Time   = 26-JUN-2021 14:57:54
Revision_Time       = 26-JUN-2021 15:03:59

Comment             = single_pulse
Data_Format         = 1D COMPLEX
Dim_Size            = 13107
X_Domain            = Proton
Dim_Title           = Proton
Dim_Units           = [ppm]
Dimensions          = X
Spectrometer        = JNM-ECZ400S/L1

Field_Strength      = 9.389766[T] (400[MHz]
X_Acq_Duration      = 2.18628096[s]
X_Domain            = Proton
X_Freq              = 399.78219838[MHz]
X_Offset            = 5[ppm]
X_Points            = 16384
X_Prescans          = 1
X_Resolution        = 0.45739775[Hz]
X_Sweep             = 7.4940048[kHz]
X_Sweep_Clipped     = 5.99520384[kHz]
Irr_Domain          = Proton
Irr_Freq            = 399.78219838[MHz]
Irr_Offset          = 5[ppm]
Tri_Domain          = Proton
Tri_Freq            = 399.78219838[MHz]
Tri_Offset          = 5[ppm]
Blanking            = 2.0[us]
Clipped             = FALSE
Scans               = 8
Total_Scans         = 8

Relaxation_Delay    = 5[s]
Recvr_Gain          = 56
Temp_Get            = 22.8[dC]
X_90_Width          = 6.6[us]
X_Acq_Time          = 2.18628096[s]
X_Angle             = 45[deg]
X_Atn               = 6.5[dB]
X_Pulse             = 3.3[us]
Irr_Mode            = Off
Tri_Mode            = Off
```

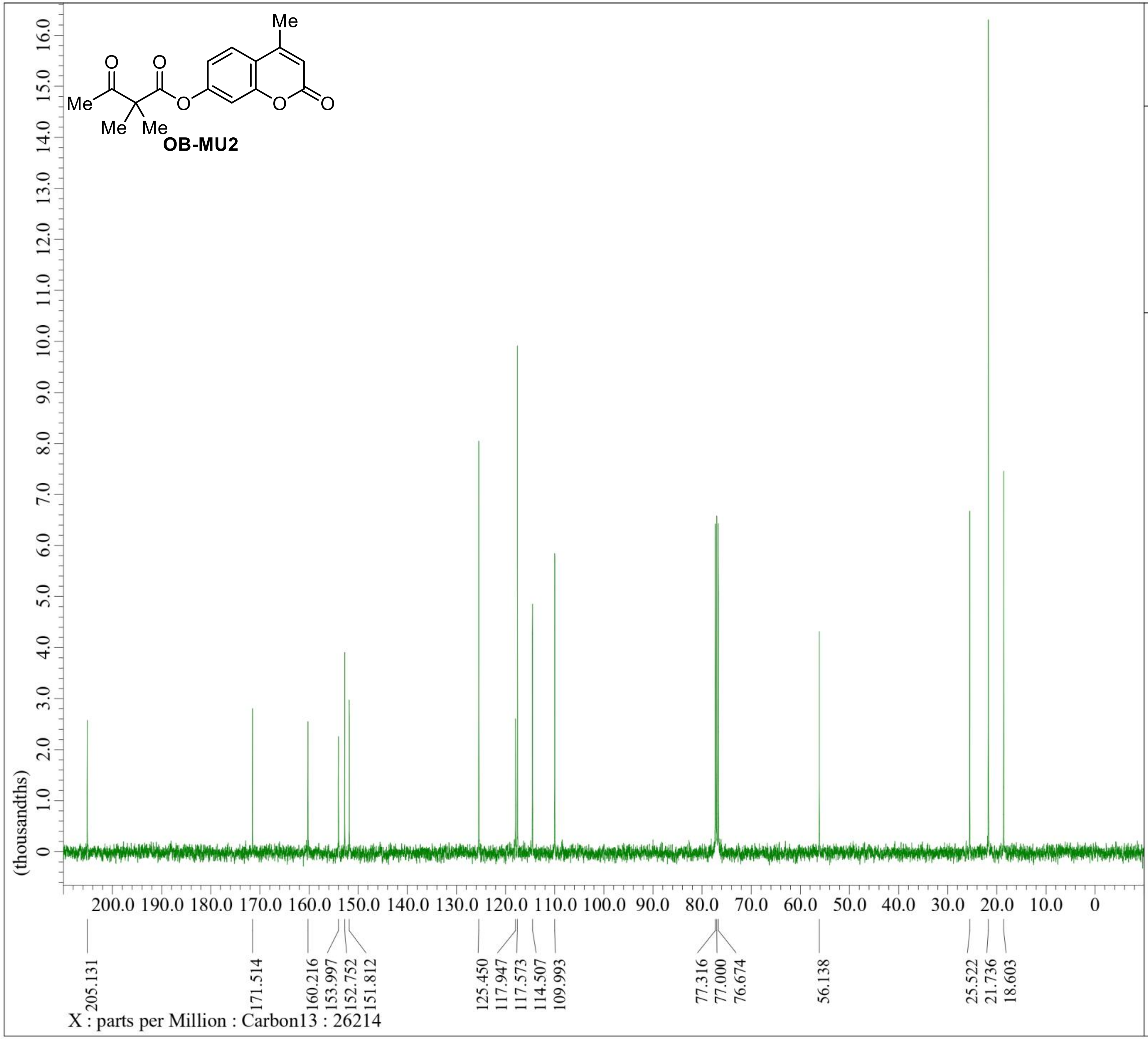

---- PROCESSING PARAMETERS ----
sexp(2.0[Hz], 0.0[s])
trapezoid(0[%], 0[%], 80[%], 100[%])
zerofill(1, TRUE)
fft(1, TRUE, TRUE)
machinephase
ppm

Filename	= 16ATA2-65-2_Carbon
Author	= delta
Experiment	= carbon_auto.jxp
Sample_Id	= 16ATA2-65-2
Solvent	= CHLOROFORM-D
Actual_Start_Time	= 26-JUN-2021 20:22:
Revision_Time	= 26-JUN-2021 20:29:
Comment	= single pulse decou
Data_Format	= 1D COMPLEX
Dim_Size	= 26214
X_Domain	= Carbon13
Dim_Title	= Carbon13
Dim_Units	= [ppm]
Dimensions	= X
Spectrometer	= JNM-ECZ400S/L1
Field_Strength	= 9.389766[T] (400[M
X_Acq_Duration	= 1.03809024[s]
X_Domain	= Carbon13
X_Freq	= 100.52530333[MHz]
X_Offset	= 100[ppm]
X_Points	= 32768
X_Prescans	= 4
X_Resolution	= 0.96330739[Hz]
X_Sweep	= 31.56565657[kHz]
X_Sweep_Clipped	= 25.25252525[kHz]
Irr_Domain	= Proton
Irr_Freq	= 399.78219838[MHz]
Irr_Offset	= 5[ppm]
Blanking	= 5.0[us]
Clipped	= FALSE
Scans	= 105
Total_Scans	= 105
Relaxation_Delay	= 2[s]
Recvr_Gain	= 50
Temp_Get	= 22.7[dC]
X_90_Width	= 10.5[us]
X_Acq_Time	= 1.03809024[s]
X_Angle	= 30[deg]
X_Atn	= 8.2[dB]
X_Pulse	= 3.5[us]
Irr_Atn_Dec	= 31.323[dB]
Irr_Atn_Dec_Calc	= 31.323[dB]
Irr_Atn_Dec_Default_Calc	= 31.323[dB]
Irr_Atn_Noe	= 31.323[dB]
Irr_Dec_Bandwidth_Hz	= 4.7826087[kHz]

date: 2022年7月22日

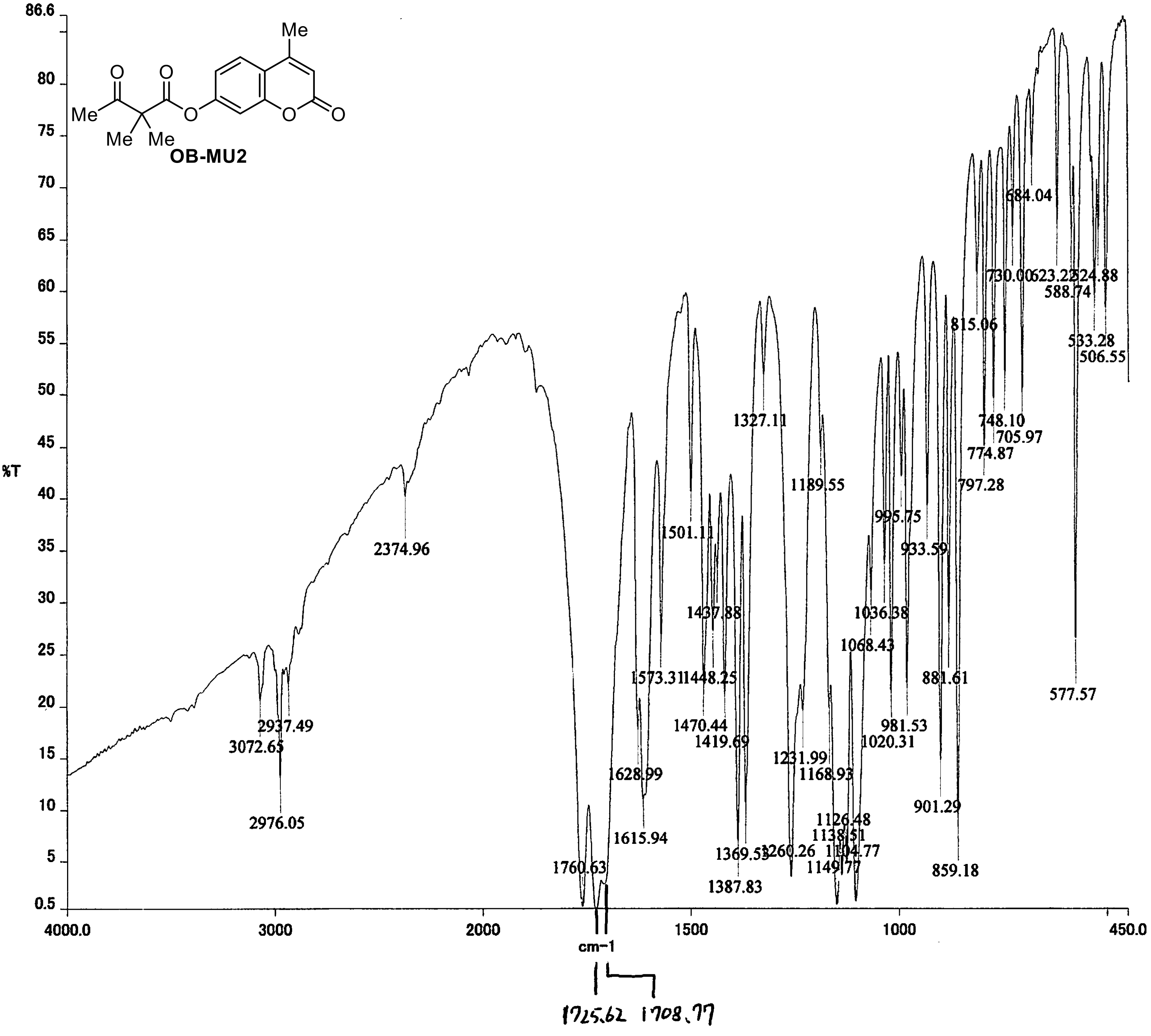

YY18HSH2-2-2 #56-68 RT: 0.30-0.35 AV: 3 SB: 4 1.00-1.11 NL: 4.04E7
F: FTMS + p ESI Full ms [100.00-1000.00]

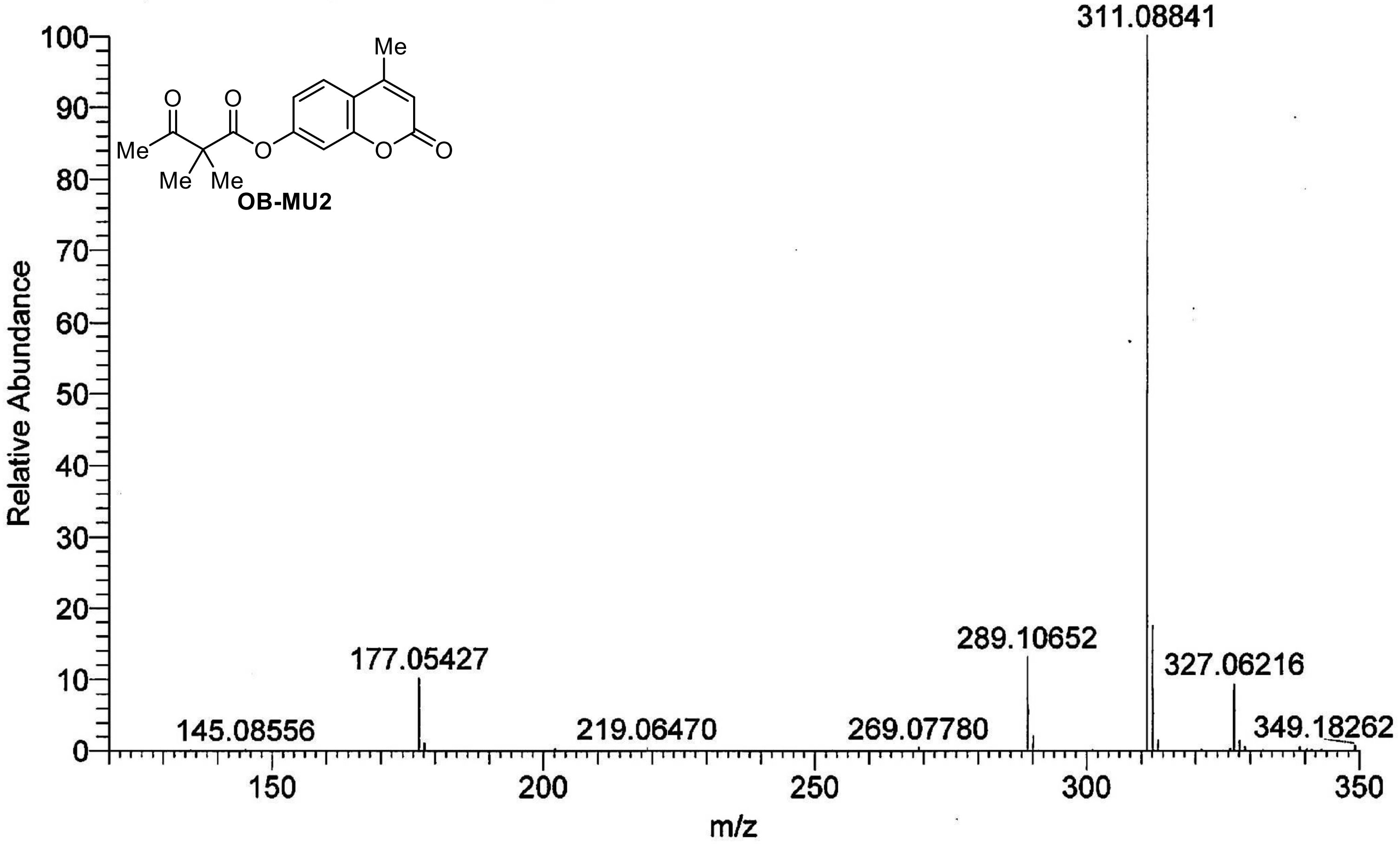

YY18HSH2-2-2#56-68 RT: 0.30-0.35 AV: 3
SB: 4 1.00-1.11
F: FTMS + p ESI Full ms [100.00-1000.00]
m/z= 288.10313-311.78142

m/z	Intensity	Relative	Theo. Mass	Delta (ppm)	Composition
289.10652	5398060.5	12.91	289.10705	-0.53	$C_{16}H_{17}O_5$
311.08841	41799176.0	100.00	311.08899	-0.59	$C_{16}H_{16}O_5Na$

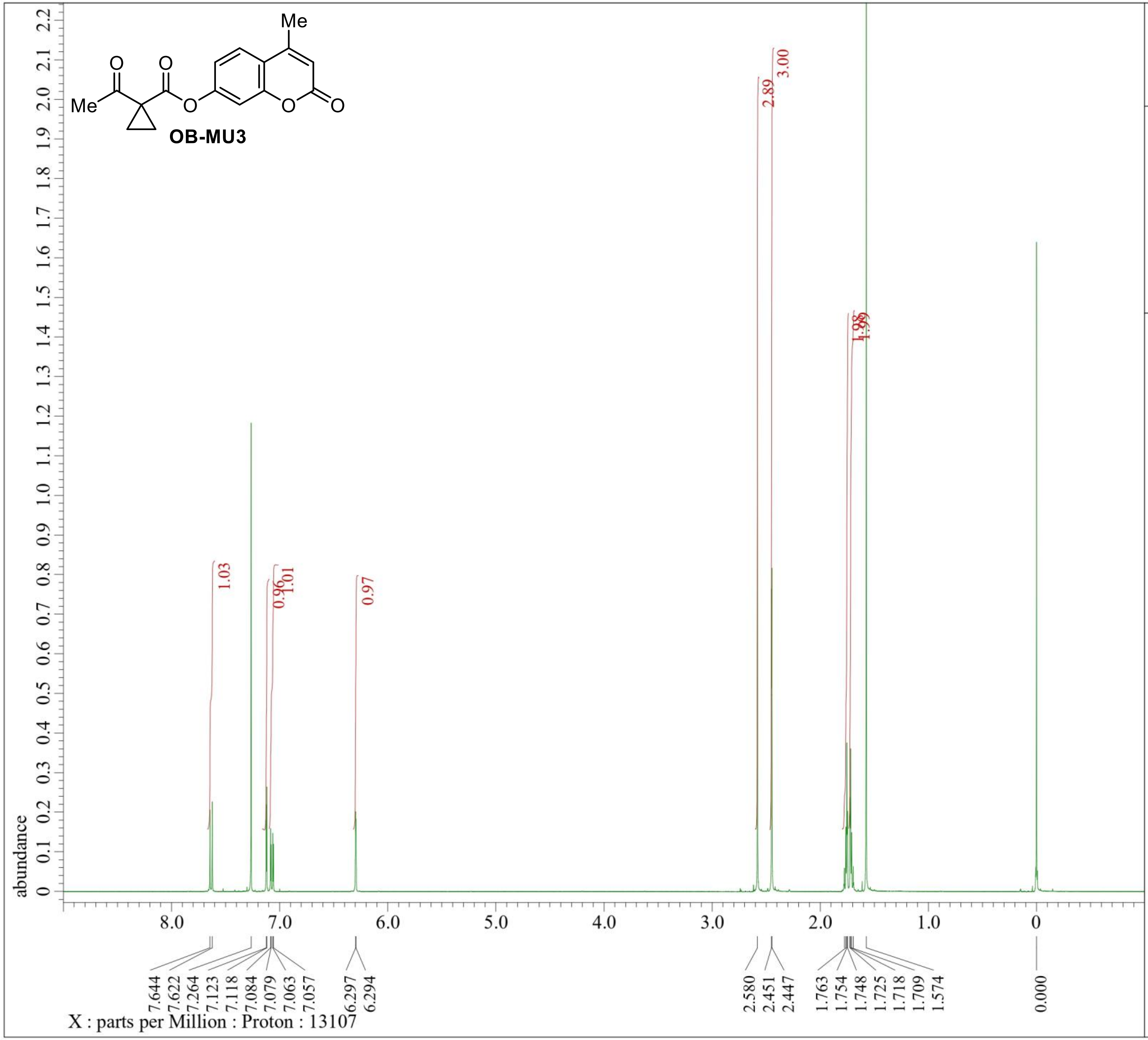

JEOL

```
---- PROCESSING PARAMETERS ----
sexp( 0.2[Hz], 0.0[s] )
trapezoid( 0[%], 0[%], 80[%], 100[%] )
zerofill( 1, TRUE )
fft( 1, TRUE, TRUE )
machinephase
ppm
phase( 2, -5, 72.95895[%] )
```

```
Filename            = 16ATA3-44-pure3_Proto
Author              = delta
Experiment          = proton_auto.jxp
Sample_Id           = 16ATA3-44-pure3
Solvent             = CHLOROFORM-D
Actual_Start_Time   = 23-JUN-2022 12:25:47
Revision_Time       = 23-JUN-2022 13:43:50

Comment             = single_pulse
Data_Format         = 1D COMPLEX
Dim_Size            = 13107
X_Domain            = Proton
Dim_Title           = Proton
Dim_Units           = [ppm]
Dimensions          = X
Spectrometer        = JNM-ECZ400S/L1

Field_Strength      = 9.389766[T] (400[MHz]
X_Acq_Duration      = 2.18628096[s]
X_Domain            = Proton
X_Freq              = 399.78219838[MHz]
X_Offset            = 5[ppm]
X_Points            = 16384
X_Prescans          = 1
X_Resolution        = 0.45739775[Hz]
X_Sweep             = 7.4940048[kHz]
X_Sweep_Clipped     = 5.99520384[kHz]
Irr_Domain          = Proton
Irr_Freq            = 399.78219838[MHz]
Irr_Offset          = 5[ppm]
Tri_Domain          = Proton
Tri_Freq            = 399.78219838[MHz]
Tri_Offset          = 5[ppm]
Blanking            = 2.0[us]
Clipped             = FALSE
Scans               = 8
Total_Scans         = 8

Relaxation_Delay    = 5[s]
Recvr_Gain          = 56
Temp_Get            = 22[dC]
X_90_Width          = 6.6[us]
X_Acq_Time          = 2.18628096[s]
X_Angle             = 45[deg]
X_Atn               = 6.5[dB]
X_Pulse             = 3.3[us]
Irr_Mode            = Off
Tri_Mode            = Off
```

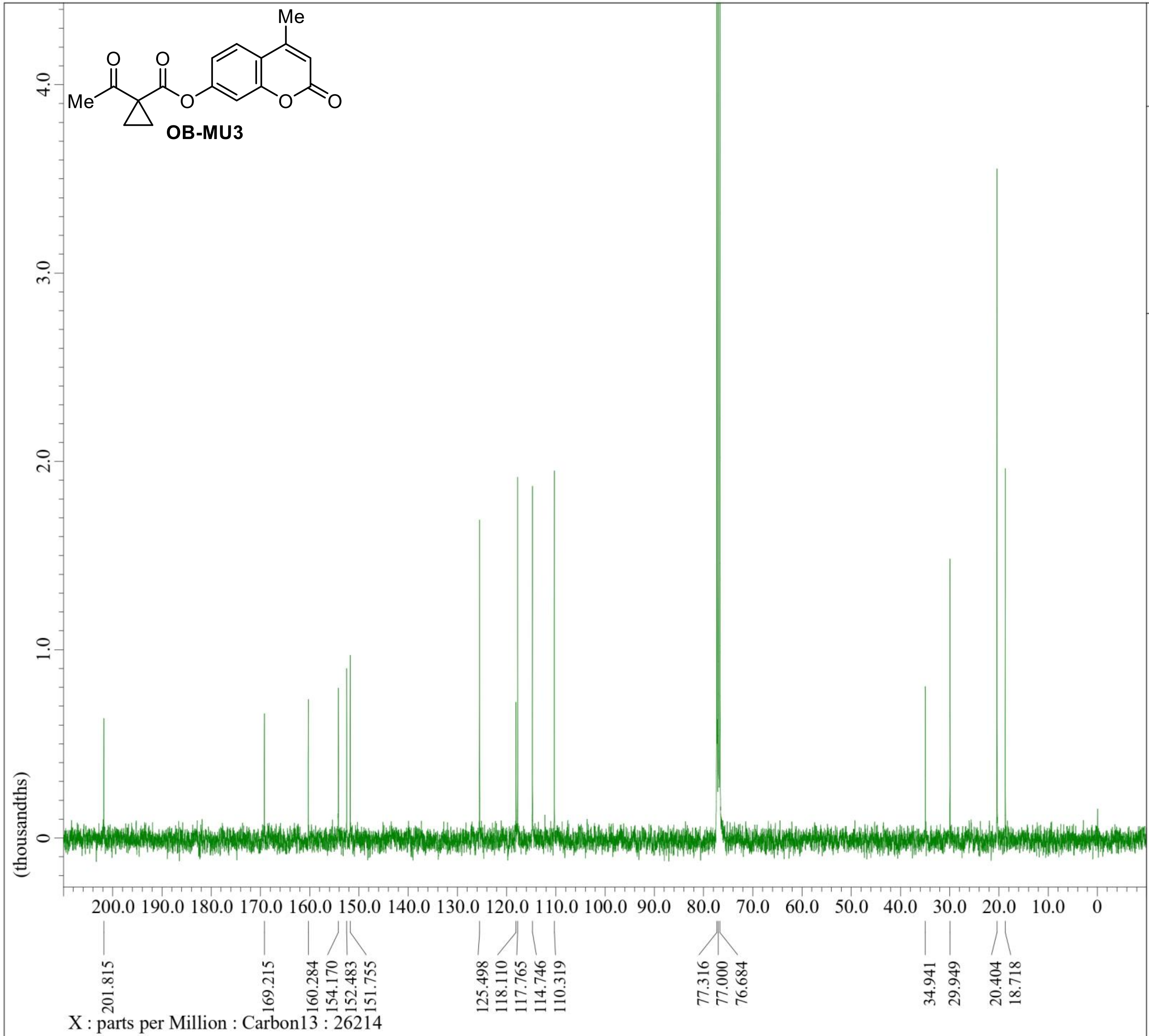


```
---- PROCESSING PARAMETERS ----
sexp( 2.0[Hz], 0.0[s] )
trapezoid( 0[%], 0[%], 80[%], 100[%] )
zerofill( 1, TRUE )
fft( 1, TRUE, TRUE )
machinephase
ppm
```

```
Filename                  = 16ATA3-44-pure_Car
Author                    = delta
Experiment                = carbon_auto.jxp
Sample_Id                 = 16ATA3-44-pure
Solvent                   = CHLOROFORM-D
Actual_Start_Time         = 21-JUN-2022 21:02:
Revision_Time             = 25-JUN-2022 11:33:

Comment                   = single pulse decou
Data_Format               = 1D COMPLEX
Dim_Size                  = 26214
X_Domain                  = Carbon13
Dim_Title                 = Carbon13
Dim_Units                 = [ppm]
Dimensions                = X
Spectrometer              = JNM-ECZ400S/L1

Field_Strength            = 9.389766[T] (400[M
X_Acq_Duration            = 1.03809024[s]
X_Domain                  = Carbon13
X_Freq                    = 100.52530333[MHz]
X_Offset                  = 100[ppm]
X_Points                  = 32768
X_Prescans                = 4
X_Resolution              = 0.96330739[Hz]
X_Sweep                   = 31.56565657[kHz]
X_Sweep_Clipped           = 25.25252525[kHz]
Irr_Domain                = Proton
Irr_Freq                  = 399.78219838[MHz]
Irr_Offset                = 5[ppm]
Blanking                  = 5.0[us]
Clipped                   = FALSE
Scans                     = 513
Total_Scans               = 513

Relaxation_Delay          = 2[s]
Recvr_Gain                = 50
Temp_Get                  = 23[dC]
X_90_Width                = 10.5[us]
X_Acq_Time                = 1.03809024[s]
X_Angle                   = 30[deg]
X_Atn                     = 8.2[dB]
X_Pulse                   = 3.5[us]
Irr_Atn_Dec               = 31.323[dB]
Irr_Atn_Dec_Calc          = 31.323[dB]
Irr_Atn_Dec_Default_Calc  = 31.323[dB]
Irr_Atn_Noe               = 31.323[dB]
Irr_Dec_Bandwidth_Hz      = 4.7826087[kHz]
```

date: 2022年7月22日

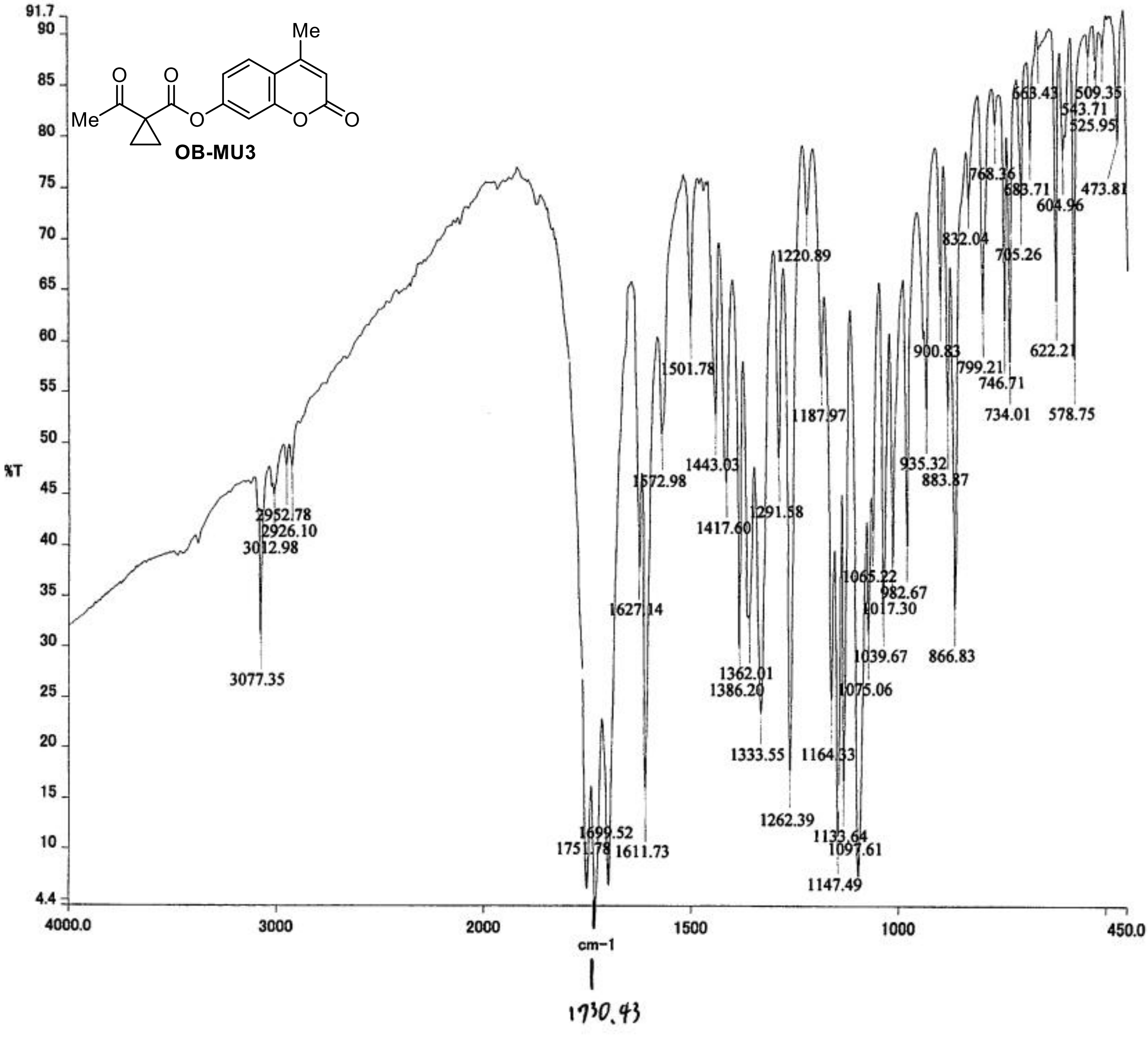

YY16ATA3-93-3 #27-32 RT: 0.62-0.70 AV: 3 SB: 3 0.40-0.52 NL: 1.55E5
T: FTMS {1,1} + p ESI Full ms [50.00-300.00]

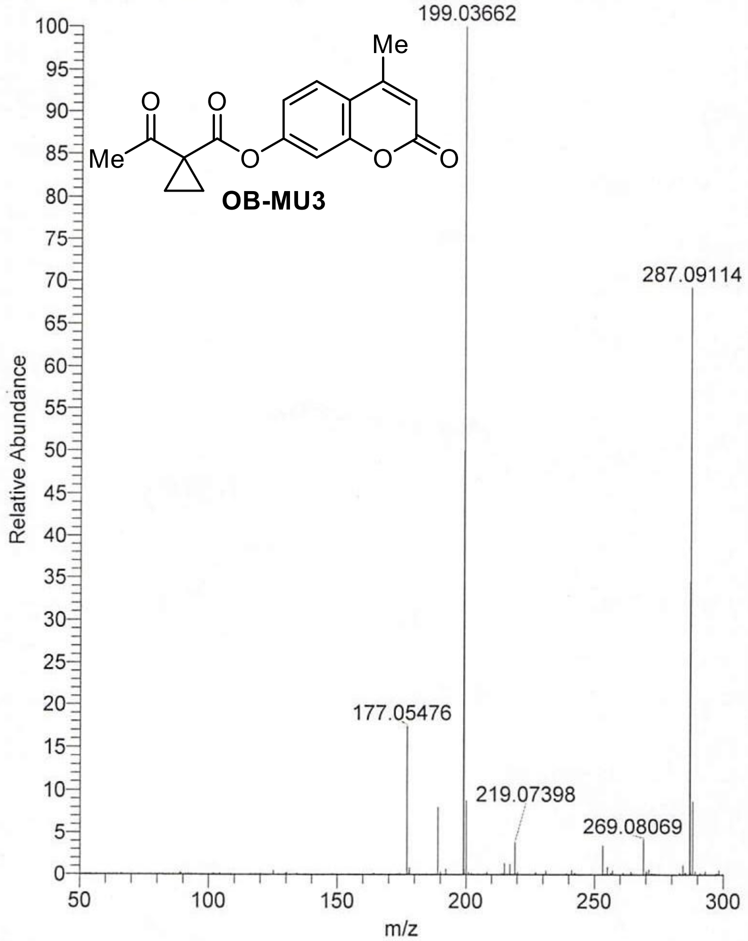

YY16ATA3-93-3 #38-40 RT: 0.84-0.88 AV: 2 SB: 3 0.40-0.52 NL: 8.93E5
T: FTMS {1,2} - p ESI Full ms [50.00-300.00]

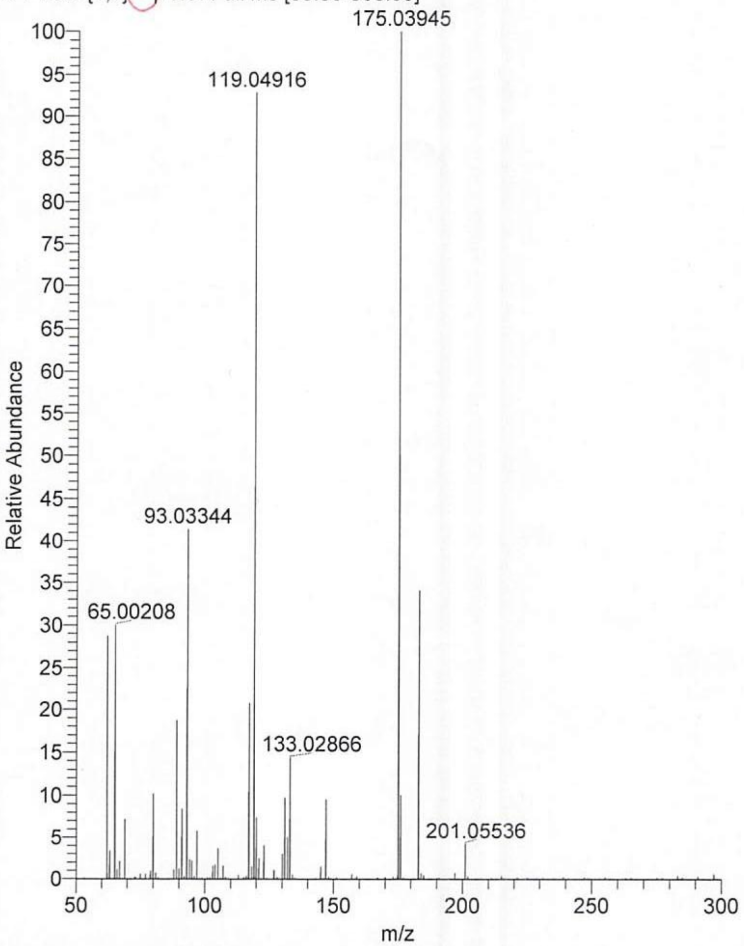

YY16ATA3-93-3#27-31 RT: 0.62-0.70 AV: 3
SB: 3 0.40-0.52
T: FTMS {1,1} + p ESI Full ms [50.00-300.00]
m/z= 164.39929-209.45230

m/z	Intensity	Relative	Theo. Mass	Delta (ppm)	Composition
177.05476	27282.1	17.24	177.05462	0.81	$C_{10}H_9O_3$
199.03662	158289.8	100.00	199.03657	0.26	$C_{10}H_8O_3$ Na

YY16ATA3-93-3#38-40 RT: 0.84-0.88 AV: 2
SB: 3 0.40-0.52
T: FTMS {1,2} - p ESI Full ms [50.00-300.00]
m/z= 170.01352-180.76481

m/z	Intensity	Relative	Theo. Mass	Delta (ppm)	Compositi
175.03945	906152.4	100.00	175.04007	-3.50	$C_{10}H_7O_3$

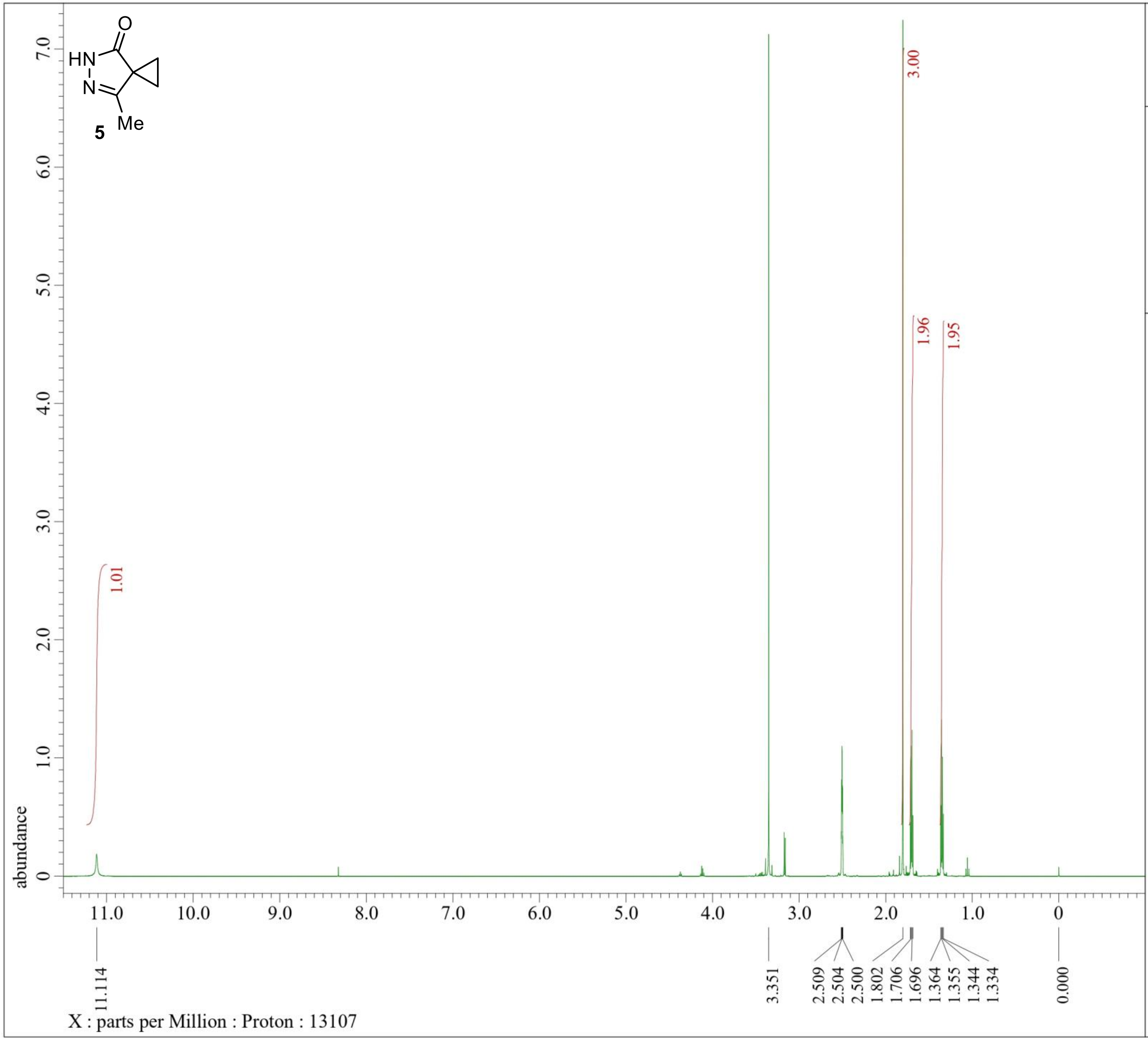

JEOL

---- PROCESSING PARAMETERS ----
sexp(0.2[Hz], 0.0[s])
trapezoid(0[%], 0[%], 80[%], 100[%])
zerofill(1, TRUE)
fft(1, TRUE, TRUE)
machinephase
ppm

以下に由来:: 16ATA3-50-3_Proton-1-1.jdf

Filename	= 16ATA3-50-3_Proton-1-
Author	= delta
Experiment	= proton_auto.jxp
Sample_Id	= 16ATA3-50-3
Solvent	= DMSO-D6
Actual_Start_Time	= 6-MAR-2022 19:33:53
Revision_Time	= 19-NOV-2024 19:52:10
Comment	= single_pulse
Data_Format	= 1D COMPLEX
Dim_Size	= 13107
X_Domain	= Proton
Dim_Title	= Proton
Dim_Units	= [ppm]
Dimensions	= X
Spectrometer	= JNM-ECZ400S/L1
Field_Strength	= 9.389766[T] (400[MHz]
X_Acq_Duration	= 2.18628096[s]
X_Domain	= Proton
X_Freq	= 399.78219838[MHz]
X_Offset	= 5[ppm]
X_Points	= 16384
X_Prescans	= 1
X_Resolution	= 0.45739775[Hz]
X_Sweep	= 7.4940048[kHz]
X_Sweep_Clipped	= 5.99520384[kHz]
Irr_Domain	= Proton
Irr_Freq	= 399.78219838[MHz]
Irr_Offset	= 5[ppm]
Tri_Domain	= Proton
Tri_Freq	= 399.78219838[MHz]
Tri_Offset	= 5[ppm]
Blanking	= 2.0[us]
Clipped	= FALSE
Scans	= 8
Total_Scans	= 8
Relaxation_Delay	= 5[s]
Recvr_Gain	= 56
Temp_Get	= 19.4[dC]
X_90_Width	= 6.6[us]
X_Acq_Time	= 2.18628096[s]
X_Angle	= 45[deg]
X_Atn	= 6.5[dB]
X_Pulse	= 3.3[us]
Irr_Mode	= Off
Tri_Mode	= Off

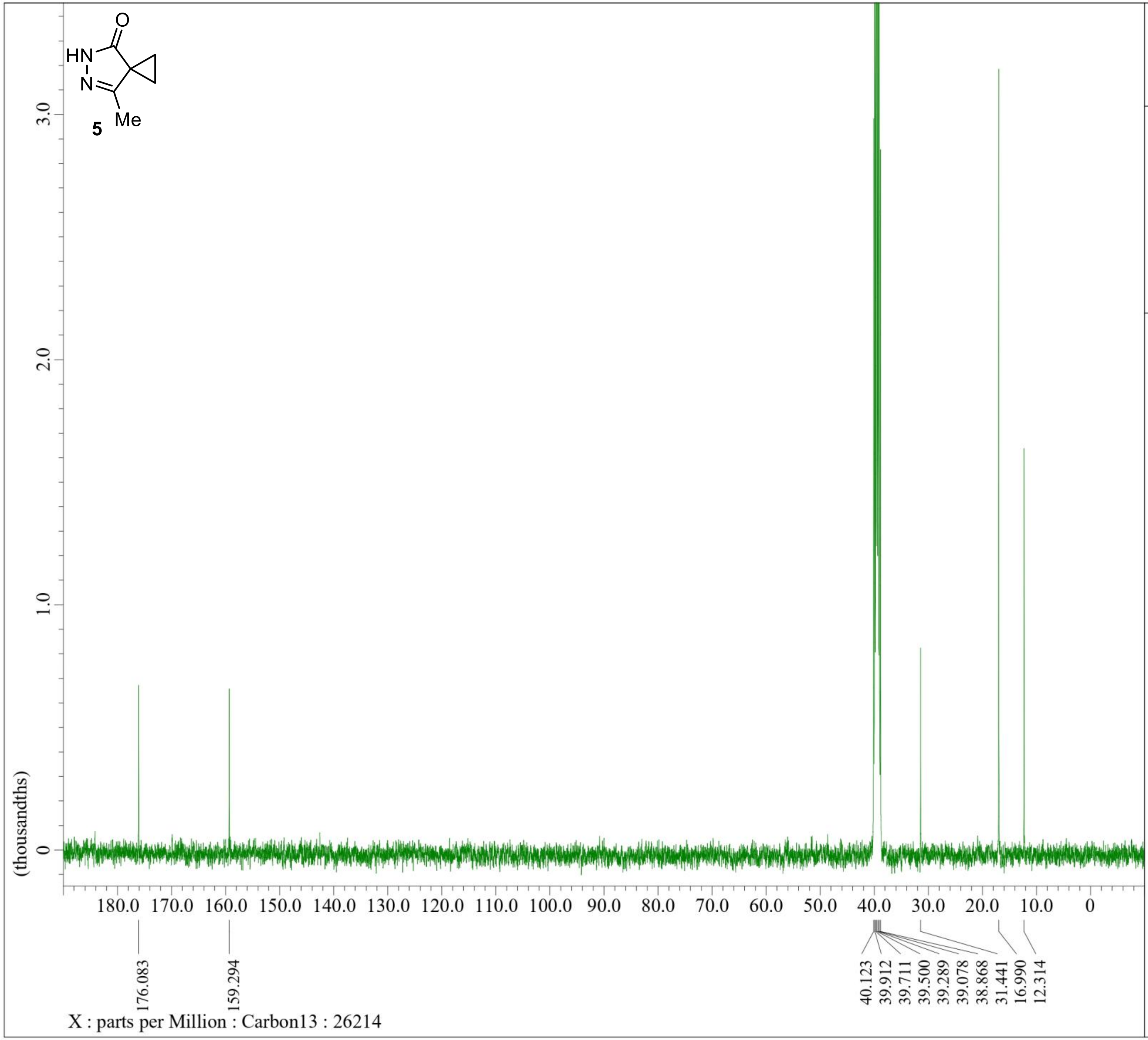


```
---- PROCESSING PARAMETERS ----
sexp( 2.0[Hz], 0.0[s] )
trapezoid( 0[%], 0[%], 80[%], 100[%] )
zerofill( 1, TRUE )
fft( 1, TRUE, TRUE )
machinephase
ppm
```

```
Filename                   = 16ATA3-50-3-13C_Ca
Author                     = delta
Experiment                 = carbon_auto.jxp
Sample_Id                  = 16ATA3-50-3-13C
Solvent                    = DMSO-D6
Actual_Start_Time          =  6-MAR-2022 20:59:
Revision_Time              =  7-MAR-2022 18:53:

Comment                    = single pulse decou
Data_Format                = 1D COMPLEX
Dim_Size                   = 26214
X_Domain                   = Carbon13
Dim_Title                  = Carbon13
Dim_Units                  = [ppm]
Dimensions                 = X
Spectrometer               = JNM-ECZ400S/L1

Field_Strength             = 9.389766[T] (400[M
X_Acq_Duration             = 1.03809024[s]
X_Domain                   = Carbon13
X_Freq                     = 100.52530333[MHz]
X_Offset                   = 100[ppm]
X_Points                   = 32768
X_Prescans                 = 4
X_Resolution               = 0.96330739[Hz]
X_Sweep                    = 31.56565657[kHz]
X_Sweep_Clipped            = 25.25252525[kHz]
Irr_Domain                 = Proton
Irr_Freq                   = 399.78219838[MHz]
Irr_Offset                 = 5[ppm]
Blanking                   = 5.0[us]
Clipped                    = FALSE
Scans                      = 1024
Total_Scans                = 1024

Relaxation_Delay           = 2[s]
Recvr_Gain                 = 50
Temp_Get                   = 19.7[dC]
X_90_Width                 = 10.5[us]
X_Acq_Time                 = 1.03809024[s]
X_Angle                    = 30[deg]
X_Atn                      = 8.2[dB]
X_Pulse                    = 3.5[us]
Irr_Atn_Dec                = 31.323[dB]
Irr_Atn_Dec_Calc           = 31.323[dB]
Irr_Atn_Dec_Default_Calc   = 31.323[dB]
Irr_Atn_Noe                = 31.323[dB]
Irr_Dec_Bandwidth_Hz       = 4.7826087[kHz]
```

YY16ATA3-93-2_221107164145 #35-37 RT: 0.80-0.85 AV: 2 SB: 3 0.40-0.52 NL: 1.84E4
T: FTMS {1,1} + p ESI Full ms [50.00-300.00]

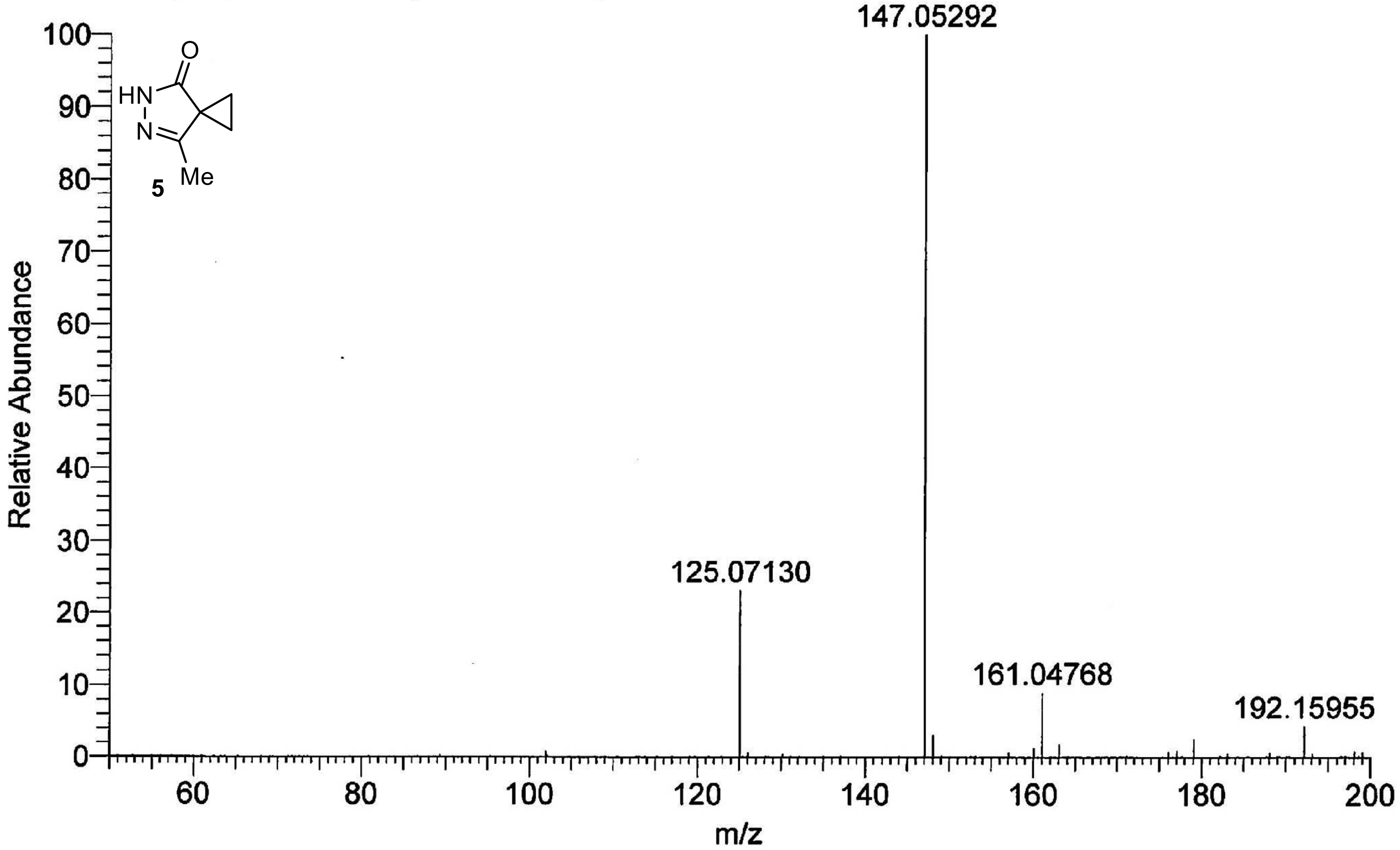

YY16ATA3-93-2_221107164145#35-37 RT: 0.80-0.85 AV: 2
SB: 3 0.40-0.52
T: FTMS {1,1} + p ESI Full ms [50.00-300.00]
m/z= 122.50831-150.16611

m/z	Intensity	Relative	Theo. Mass	Delta (ppm)	Composition
125.07130	4260.8	22.88	125.07094	2.91	$C_6H_9ON_2$
147.05292	18624.5	100.00	147.05288	0.25	$C_6H_8ON_2Na$

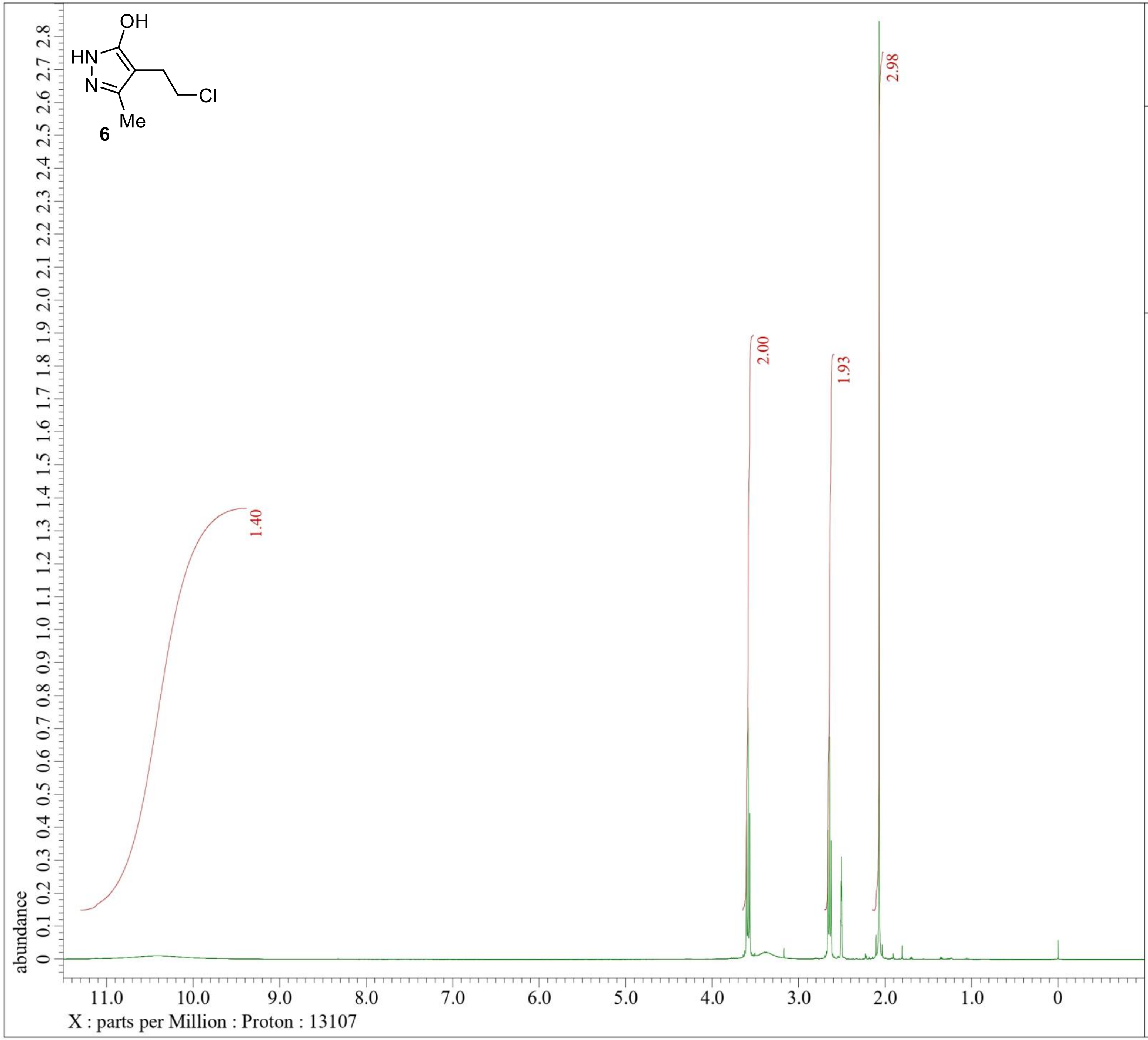

JEOL

---- PROCESSING PARAMETERS ----
sexp(0.2[Hz], 0.0[s])
trapezoid(0[%], 0[%], 80[%], 100[%])
zerofill(1, TRUE)
fft(1, TRUE, TRUE)
machinephase
ppm

Filename	= 16ATA3-50-4_Proton-1-
Author	= delta
Experiment	= proton_auto.jxp
Sample_Id	= 16ATA3-50-4
Solvent	= DMSO-D6
Actual_Start_Time	= 6-MAR-2022 19:39:32
Revision_Time	= 6-MAR-2022 20:23:37
Comment	= single_pulse
Data_Format	= 1D COMPLEX
Dim_Size	= 13107
X_Domain	= Proton
Dim_Title	= Proton
Dim_Units	= [ppm]
Dimensions	= X
Spectrometer	= JNM-ECZ400S/L1
Field_Strength	= 9.389766[T] (400[MHz]
X_Acq_Duration	= 2.18628096[s]
X_Domain	= Proton
X_Freq	= 399.78219838[MHz]
X_Offset	= 5[ppm]
X_Points	= 16384
X_Prescans	= 1
X_Resolution	= 0.45739775[Hz]
X_Sweep	= 7.4940048[kHz]
X_Sweep_Clipped	= 5.99520384[kHz]
Irr_Domain	= Proton
Irr_Freq	= 399.78219838[MHz]
Irr_Offset	= 5[ppm]
Tri_Domain	= Proton
Tri_Freq	= 399.78219838[MHz]
Tri_Offset	= 5[ppm]
Blanking	= 2.0[us]
Clipped	= FALSE
Scans	= 8
Total_Scans	= 8
Relaxation_Delay	= 5[s]
Recvr_Gain	= 46
Temp_Get	= 19.5[dC]
X_90_Width	= 6.6[us]
X_Acq_Time	= 2.18628096[s]
X_Angle	= 45[deg]
X_Atn	= 6.5[dB]
X_Pulse	= 3.3[us]
Irr_Mode	= Off
Tri_Mode	= Off

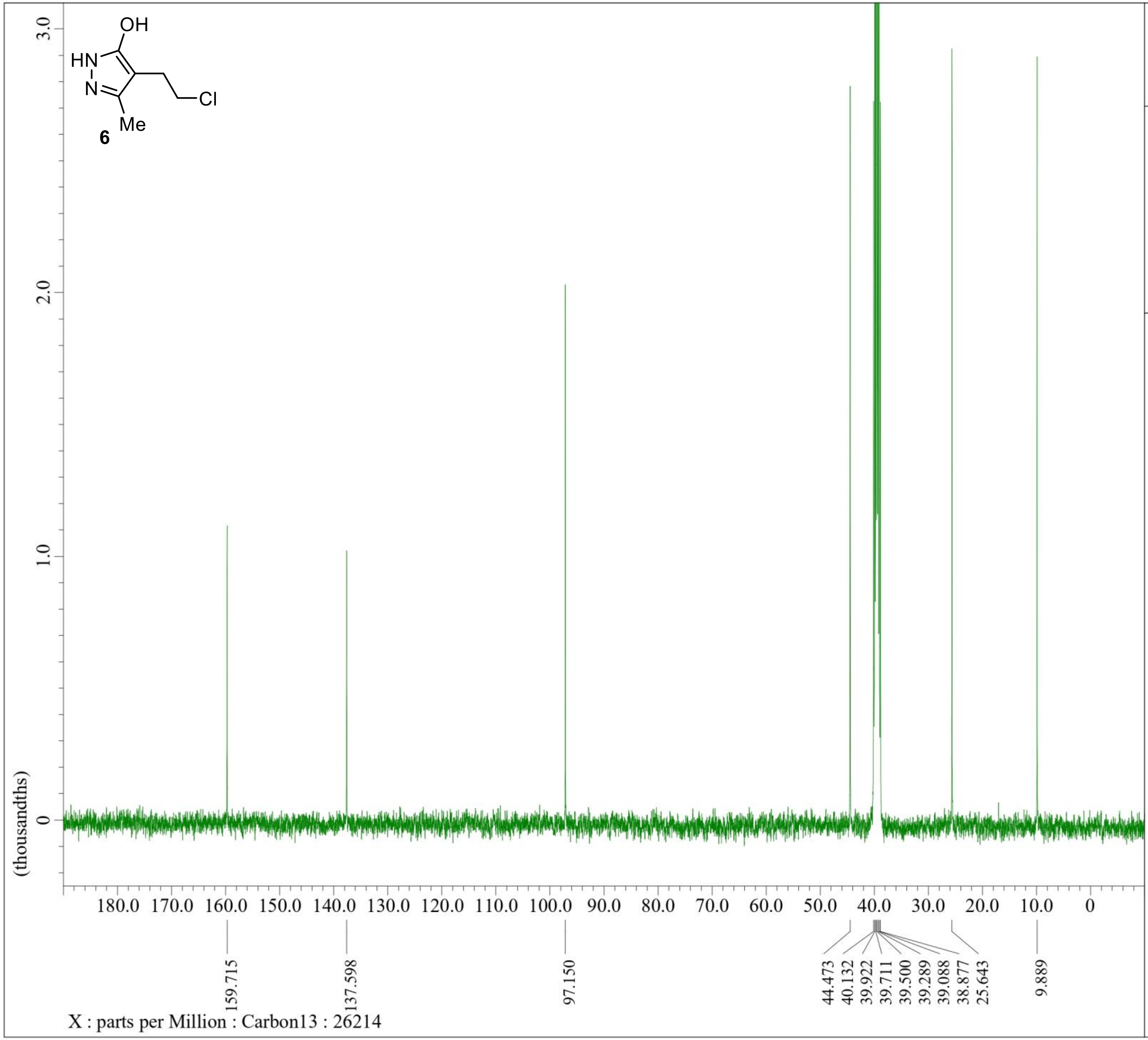


```
---- PROCESSING PARAMETERS ----
sexp( 2.0[Hz], 0.0[s] )
trapezoid( 0[%], 0[%], 80[%], 100[%] )
zerofill( 1, TRUE )
fft( 1, TRUE, TRUE )
machinephase
ppm
phase( -2, 0, 50[%] )
```

```
Filename                   = 16ATA3-50-4-13C_Ca
Author                     = delta
Experiment                 = carbon_auto.jxp
Sample_Id                  = 16ATA3-50-4-13C
Solvent                    = DMSO-D6
Actual_Start_Time          =  6-MAR-2022 21:55:
Revision_Time              =  7-MAR-2022 18:54:

Comment                    = single pulse decou
Data_Format                = 1D COMPLEX
Dim_Size                   = 26214
X_Domain                   = Carbon13
Dim_Title                  = Carbon13
Dim_Units                  = [ppm]
Dimensions                 = X
Spectrometer               = JNM-ECZ400S/L1

Field_Strength             = 9.389766[T] (400[M
X_Acq_Duration             = 1.03809024[s]
X_Domain                   = Carbon13
X_Freq                     = 100.52530333[MHz]
X_Offset                   = 100[ppm]
X_Points                   = 32768
X_Prescans                 = 4
X_Resolution               = 0.96330739[Hz]
X_Sweep                    = 31.56565657[kHz]
X_Sweep_Clipped            = 25.25252525[kHz]
Irr_Domain                 = Proton
Irr_Freq                   = 399.78219838[MHz]
Irr_Offset                 = 5[ppm]
Blanking                   = 5.0[us]
Clipped                    = FALSE
Scans                      = 1024
Total_Scans                = 1024

Relaxation_Delay           = 2[s]
Recvr_Gain                 = 50
Temp_Get                   = 19.8[dC]
X_90_Width                 = 10.5[us]
X_Acq_Time                 = 1.03809024[s]
X_Angle                    = 30[deg]
X_Atn                      = 8.2[dB]
X_Pulse                    = 3.5[us]
Irr_Atn_Dec                = 31.323[dB]
Irr_Atn_Dec_Calc           = 31.323[dB]
Irr_Atn_Dec_Default_Calc   = 31.323[dB]
Irr_Atn_Noe                = 31.323[dB]
Irr_Dec_Bandwidth_Hz       = 4.7826087[kHz]
```

C:\Xcalibur\...\YY16ATA3-93-3-2 11/8/2022 9:07:22 AM

YY16ATA3-93-3-2 #35-37 RT: 0.78-0.83 AV: 2 SB: 3 0.41-0.52 NL: 6.72E4
T: FTMS {1,1} + p ESI Full ms [50.00-300.00]

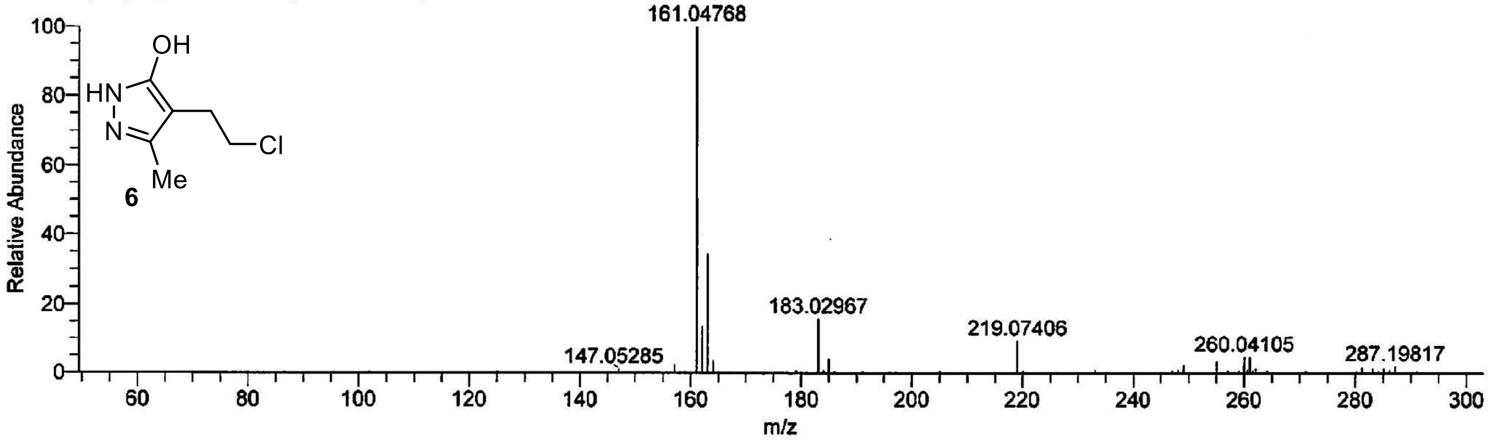

YY16ATA3-93-3-2 #35-37 RT: 0.78-0.83 AV: 2 SB: 3 0.41-0.52 NL: 6.72E4
T: FTMS {1,1} + p ESI Full ms [50.00-300.00]

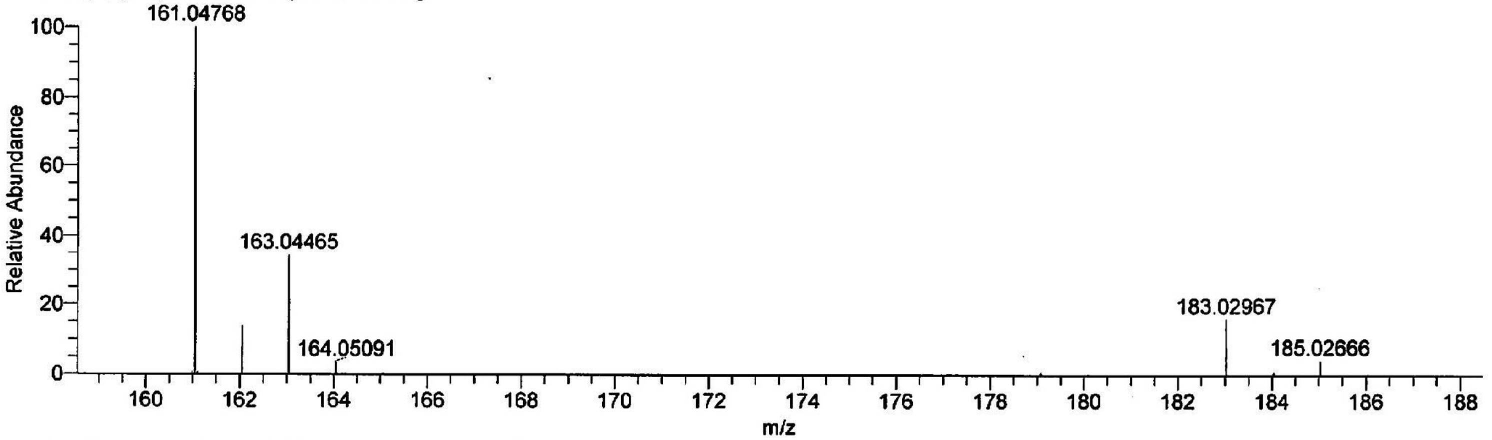

YY16ATA3-93-3-2#35-37 RT: 0.78-0.83 AV: 2
SB: 3 0.41-0.52
T: FTMS {1,1} + p ESI Full ms [50.00-300.00]
m/z= 158.54604-188.46439

m/z	Intensity	Relative	Theo. Mass	Delta (ppm)	Composition
161.04768	68974.2	100.00	161.04762	0.38	$C_6H_{10}ON_2Cl$
163.04465	23587.0	34.20	163.04467	-0.08	$C_6H_{10}ON_2{}^{37}Cl$

YY16ATA3-93-3-2#35-37 RT: 0.78-0.83 AV: 2
SB: 3 0.41-0.52
T: FTMS {1,1} + p ESI Full ms [50.00-300.00]
m/z= 182.62485-185.30855

m/z	Intensity	Relative	Theo. Mass	Delta (ppm)	Composition
183.02967	10720.1	100.00	183.02956	0.60	$C_6H_9ON_2ClNa$
185.02666	2779.6	25.93	185.02661	0.25	$C_6H_9ON_2{}^{37}ClNa$
